# Distinct neural representational changes following cross-format number tutoring in children with mathematical difficulties

**DOI:** 10.1038/s41539-025-00345-y

**Published:** 2025-08-13

**Authors:** Yunji Park, Yuan Zhang, Flora Schwartz, Teresa Iuculano, Hyesang Chang, Vinod Menon

**Affiliations:** 1 Department of Psychiatry & Behavioral Sciences, Stanford University, Stanford, CA USA; 2 CLLE, Université de Toulouse & CNRS, Toulouse, France; 3 Centre National de la Recherche Scientifique & Université Paris Sorbonne, Paris, France; 4 Department of Neurology and Neurological Sciences, Stanford University, Stanford, CA USA; 5 Stanford Neuroscience Institute, Stanford University, Stanford, California, CA USA; 6 Symbolic Systems Program, Stanford University, Stanford, California, CA USA

**Keywords:** Human behaviour, Problem solving, Human behaviour

## Abstract

Children with mathematical difficulties (MD) often struggle to connect abstract numerical symbols with corresponding nonsymbolic quantities, a foundational skill for mathematical development. We evaluated a 4-week personalized cross-format number (CFN) tutoring program designed to strengthen these symbolic–nonsymbolic mappings in children with MD aged 7–10 years. CFN tutoring was associated with significant improvements in numerical and arithmetic fluency. Neural representational similarity (NRS) analysis revealed that deficient cross-format NRS in children with MD was normalized following tutoring, aligning with pre-tutoring levels of typically-developing (TD) peers. This normalization was most pronounced in parietal and parahippocampal regions known to support quantity and spatial representation. We observed a distinctive pattern of neural plasticity across groups—children with MD showed increased cross-format NRS following tutoring, while TD children showed a decrease—suggesting a nonlinear, skill-dependent plasticity. These findings underscore the need for developmentally tailored interventions to support children with MD through targeted, evidence-based strategies.

## Introduction

Numerical fluency, defined as the ease and speed with which individuals understand and manipulate symbolic and nonsymbolic numerical quantities^1–7^, is essential for developing mathematical skills in early childhood^4,8–11^. Deficiencies in numerical fluency are linked to mathematical difficulties (MD), which affect up to 14% of children^12^. These difficulties pose considerable hurdles to educational and developmental progress^13–15^. Despite this, many interventions designed to improve numerical fluency are not specifically tailored to meet the needs of children with MD^16–22^. Furthermore, their effectiveness in enhancing broader mathematical skills remain inconclusive^21,23,24^. One leading theory is that MD stems from difficulties in bridging *nonsymbolic* representations (such as sets of objects) with *symbolic* forms (such as Arabic numerals)^14,25–30^.

Interventions focusing on enhancing this cross-format mapping could potentially improve math abilities in children with MD. Despite its importance, there is a notable absence of research on how specific training affects the neural underpinnings of cross-format numerical integration in children with varying levels of math proficiency (see Supplementary Table 1 for a summary of prior studies). This gap highlights a critical need for research that explores how targeted training can strengthen the neural connections between different formats of number representation. Addressing this need could lead to more effective, customized interventions for children with MD, ultimately bridging the gap in our understanding of numerical cognition and its impact on mathematical learning.

Here, for the first time, we elucidate the neurocognitive mechanisms underpinning the efficacy of a cross-format number (CFN) tutoring program specifically designed to improve weak numerical understanding in children with MD. Deficits in the ability to accurately associate symbolic numbers with their corresponding nonsymbolic quantities^2,7,31^ are a hallmark of MD^14,25–30^. We focus on how children with MD process and map numerical information across these formats. Theoretical models propose that in typically developing (TD) children, early learning stages involve associating concrete objects with abstract numerical symbols to facilitate symbolic numerical problem-solving^32,33^. As TD children advance in proficiency, a phenomenon known as ‘symbolic estrangement’ often emerges, wherein distinct neural representations for different number formats develop, reflecting increased symbolic numerical independence^34–39^.

The symbolic estrangement account is supported by functional neuroimaging studies with adults^34,40,41^ and children^35,36^. For instance, higher arithmetic proficiency correlates with greater dissociation between symbolic and nonsymbolic neural representations of numbers in the parietal cortex among individuals^34^. In adults, aligned with the hypothesis that symbolic and nonsymbolic numbers operate independently, the neural similarity space for nonsymbolic numbers aligns with numerical ratios, while that for symbolic numbers correlates with their frequency of occurrence^40^. Additionally, distinct neural pathways in the parietal cortex represent symbolic and nonsymbolic numbers, further evidencing their dissociation in adults^41^.

In one of the first studies to investigate this question in a developmental setting, Schwartz and colleagues (2021) found that higher neural similarity between symbolic and nonsymbolic numbers across various brain regions was associated with better arithmetic skills in children aged 7–10, but not in young adults (ages 14–21), suggesting that dissociation between the two formats might manifest later in development^35^. Similarly, Nakai and colleagues (2023) observed that neural overlap between symbolic and nonsymbolic numbers in the parietal cortex diminished from early to later stages of formal education^36^. However, previous studies have not focused on children with mathematical difficulties, leaving it unclear how the typical developmental progression—from initial integration to subsequent separation of numerical representations—may be disrupted or delayed in this population.

Previous investigations have focused on the approximate number system, an innate cognitive system enabling approximate comparisons of nonsymbolic quantities^42–44^, as a potential approach for remediating numerical deficits in MD. Despite its intuitive appeal, the efficacy of interventions targeting the approximate number system has been debated, with recent findings suggesting limited impact on enhancing nonsymbolic quantity discrimination or boosting symbolic math skills^45–48^. Moreover, a comprehensive understanding of number knowledge —which includes the precise alignment of symbolic numbers with their nonsymbolic representations—is increasingly recognized as critical yet deficient in individuals with MD^14,25–30^. Distinct from previous interventions that centered on nonsymbolic number training, the CFN tutoring program aims to improve the integration of symbolic number concepts with their nonsymbolic counterparts. This approach addresses a critical aspect of numerical fluency not sufficiently covered in earlier studies^45–47^, enhancing both the depth and breadth of numerical understanding in children with mathematical difficulties.

**Fig. 1:**
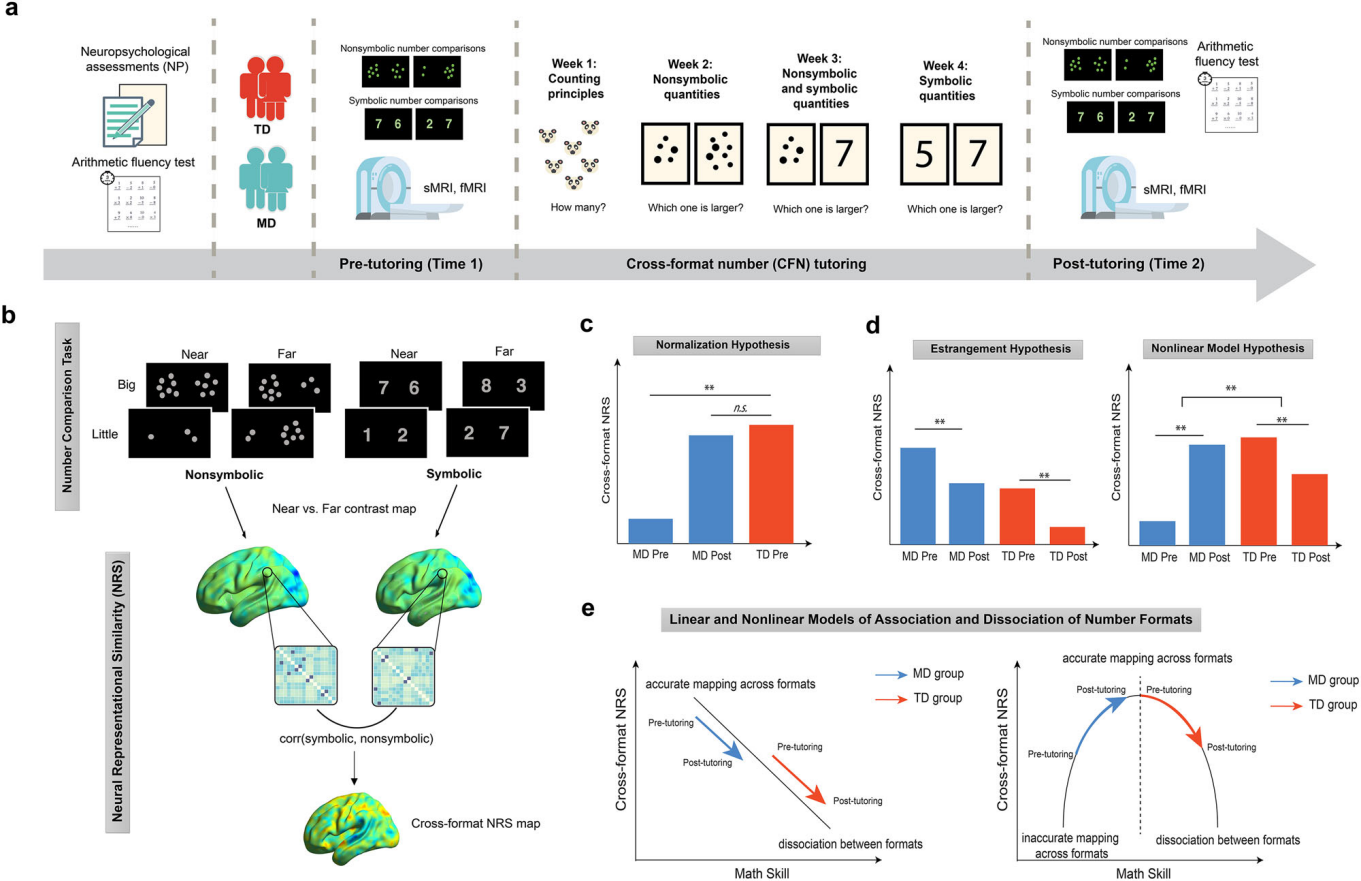
Study design, analysis steps, and key hypotheses. **a** Study design. Children with mathematical difficulties (MD) and typically developing (TD) peers underwent pre-tutoring assessments and fMRI scans, followed by a 4-week cross-format number (CFN) tutoring program and post-tutoring assessments and fMRI scans. **b** fMRI task and analysis. Participants performed nonsymbolic and symbolic number comparison tasks during fMRI scans. We examined whether CFN tutoring induced changes in cross-format neural representational similarity (NRS) between nonsymbolic and symbolic numbers. **c** Neural normalization hypothesis. We posited that CFN tutoring would enhance cross-format NRS in children with MD at post-tutoring, reaching levels similar to TD peers at pre-tutoring. **d** Linear and nonlinear model hypotheses. Expected outcomes illustrate similar or distinct effects of CFN tutoring across MD and TD groups. A linear model predicts universal decreases in cross-format NRS across MD and TD groups, consistent with the symbolic estrangement account, which suggests increases in dissociation between symbolic and nonsymbolic number representations across math skill development. Alternatively, a nonlinear model predicts divergent neural plasticity: children with MD would exhibit increased cross-format NRS following tutoring, reflecting enhanced mapping between symbolic and nonsymbolic number representations; in contrast, TD children would show decreased cross-format similarity post-tutoring, reflecting a shift toward more distinct symbolic number representations. **e** Linear and nonlinear models of association and dissociation of number formats. Schematic models are presented to illustrate potentially similar or distinct trajectories of changes in cross-format NRS as a function of short-term learning in response to tutoring in MD and TD groups. ** hypothetical significant difference. *n.s*. hypothetical non-significant difference.

Our CFN tutoring program was designed to improve the ability to map abstract numerical symbols to concrete quantities in children with MD^39,49,50^, building on previous behavioral and neuroimaging research^16,21,32,51–53^. This tutoring program consisted of individualized sessions where children progressively learned to associate numbers within and across symbolic and nonsymbolic formats across 4 weeks (Fig. 1a). We examined behavioral performance and multivariate patterns of brain activity across symbolic and nonsymbolic number comparison tasks, administered to a well-matched sample of 7–10-year-old children with MD and TD controls during functional magnetic resonance imaging (fMRI) sessions before and after CFN tutoring. Additionally, we assessed the broader impact of CFN tutoring on standardized arithmetic fluency test performance in these children. This test aimed to determine if the tutoring program could also enhance broader math problem-solving skills in children with MD, which would indicate transfer of learning to other types of mathematical skills that were not directly trained (Fig. 1a).

Crucially, we employed neural representational similarity (NRS) analysis^54–57^ to probe how training influences the neural mapping between symbolic and nonsymbolic representations of quantity in children with MD (Fig. 1b). NRS analysis is particularly well-suited for examining how children represent numbers in different formats, a crucial aspect of their mathematical skill development. Cross-format NRS between symbolic and nonsymbolic numbers offers several advantages in this context. First, it allows for precise analysis of the neural mechanisms underlying shared representations across symbolic and nonsymbolic number formats. Understanding such cross-format neural mapping is crucial, as difficulties in integrating the two number formats are often implicated in MD^14,25–30^. Second, cross-format NRS analysis sheds light on brain plasticity in response to intervention designed to integrate the numerical formats. Third, NRS analysis can identify subtle yet significant shifts in neural representations at a fine spatial scale^58–61^.

Our multivariate analysis goes beyond traditional single-voxel univariate methods, offering deeper insights into the complex relationships between neural representations and numerical tasks^62–66^. Previous research by Schwartz et al. (2021) illustrated developmental differences in cross-format NRS and arithmetic skills^35^, while Park et al. (2024) demonstrated that CFN tutoring induces a dissociation of symbolic numbers from nonsymbolic representations of quantity in children^39^. However, these studies did not specifically examine how CFN tutoring impacts the neural mappings between symbolic and nonsymbolic numbers in children with MD. Our study aims to fill this gap by detailing how CFN tutoring influences these neural connections in children with MD compared to their TD peers. This focus is crucial for developing effective, evidence-based educational interventions tailored to remediate specific learning difficulties and enhance mathematical abilities in children with MD.

We had three primary objectives. Our first objective was to evaluate whether the 4-week CFN tutoring program could normalize weak levels of similarity in cross-format numerical processing in children with MD, bringing them closer to the pre-tutoring baseline levels of their TD peers. We hypothesized that CFN tutoring would induce a more similar processing across symbolic and nonsymbolic number discrimination in children with MD. Furthermore, we aimed to assess whether the tutoring would also lead to gains in arithmetic fluency. We reasoned that if CFN tutoring was effective in facilitating transfer of learning, children with MD would show improvements in broader mathematical problem-solving abilities, reaching the performance of TD children before tutoring.

Our second objective was to determine whether CFN tutoring could facilitate *neural normalization* of cross-format similarity in number representations in children with MD. Specifically, we determined whether cross-format NRS between symbolic and nonsymbolic numbers (Fig. 1b) of children with MD at post-tutoring would align with those of TD children at pre-tutoring. We tested the hypothesis that CFN tutoring would normalize cross-format NRS in children with MD, bringing it to the level observed in TD children prior to tutoring (Fig. 1c).

The third objective of our study was to investigate whether CFN tutoring alters cross-format similarity in numerical processing and neural representational patterns differently between children with MD and their TD peers. We formulated two competing hypotheses (Fig. 1d, e). First, drawing on the symbolic estrangement account^34–36^, a linear model predicts a decline in cross-format NRS in both MD and TD groups, aligning with an increasing dissociation of symbolic and nonsymbolic number representations. Alternatively, a nonlinear model hypothesizes that cross-format NRS initially increases during early learning stages before decreasing as symbolic mathematics proficiency develops. We expected that children with MD would exhibit increased cross-format similarity in numerical processing and NRS as they improve in mapping between symbolic and nonsymbolic numbers through tutoring. In contrast, TD children, likely achieving accurate cross-format mapping earlier, might show decreased cross-format similarity post-tutoring, reflecting a shift toward more distinct symbolic number representations. Testing these competing hypotheses would highlight and distinguish the patterns of cross-format number representation plasticity between children with MD and their TD peers.

Our findings advance knowledge of the mechanisms by which CFN tutoring facilitates behavioral and neural normalization in children with MD. By testing our hypothesized model, the present study not only highlights the effectiveness of targeted intervention but also provides a window into discovering distinct patterns of learning and neural plasticity across children with diverse cognitive abilities. Importantly, these insights may help guide the development of customized interventions tailored for children with MD.

## Results

### CFN tutoring normalizes weak cross-format similarity in numerical processing in children with MD

The first objective of our study was to evaluate the efficacy of a 4-week CFN tutoring program in enhancing cross-format similarity in numerical processing in children with MD to the level of their TD peers before tutoring (see Fig. 1a and Methods). As a measure of cross-format similarity in numerical processing across symbolic and nonsymbolic formats, children’s *between-format dissimilarity* was computed as the absolute difference in efficiency scores (accuracy/median RT) between symbolic and nonsymbolic number comparison tasks ( | Symbolic - Nonsymbolic | ). Lower scores on between-format dissimilarity represented more similar processing of different number formats.

**Fig. 2:**
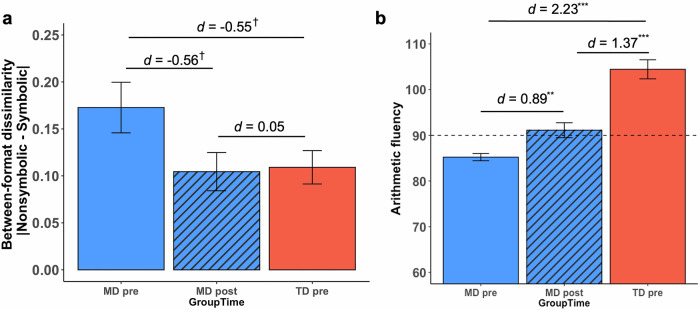
Behavioral normalization of weak cross-format similarity and transfer of learning to arithmetic skills in children with MD following cross-format number tutoring. **a** Behavioral normalization of weak cross-format similarity between nonsymbolic and symbolic numbers in children with MD. As a measure of cross-format similarity in numerical processing, a metric of *between-format dissimilarity* was obtained as the absolute difference between nonsymbolic and symbolic number comparison task efficiency. Lower scores on between-format dissimilarity represented higher cross-format similarity in numerical processing. Pre-tutoring, the MD group had higher behavioral between-format dissimilarity (lower cross-format similarity in numerical processing), compared to TD peers. Post-tutoring, this dissimilarity was reduced, aligning with pre-tutoring TD levels. **b** Transfer of learning to arithmetic fluency in children with MD. Tutoring led to a significant transfer of learning to arithmetic fluency in the MD group, narrowing the pre-existing gap with TD peers. ^†^*p* < 0.10, ^**^p < 0.01, ^***^p < 0.001, *d* = Cohen’s *d*. MD = children with mathematical difficulties, TD = typically developing children.

At pre-tutoring, the MD group displayed a higher level of between-format dissimilarity – indicative of weak cross-format similarity in numerical processing – compared to the TD group (*t*(51) = −1.99, *p* = 0.052, Cohen’s *d* = −0.55) (Fig. 2a). Similarly, between-format dissimilarity assessed using reaction times was also significantly higher in the MD group compared to the TD group (*t*(51) = −2.19, *p* = 0.033, Cohen’s *d* = −0.60; Supplementary Results). However, no significant group difference in between-format dissimilarity was observed when comparing post-tutoring children with MD with pre-tutoring TD children (*ps* > 0.864, |Cohen’s *ds* | < 0.05).

Additional analyses revealed that these changes in between-format dissimilarity with efficiency scores were largely due to changes in reaction times (Supplementary Results and Supplementary Fig. 1). Our results demonstrate that CFN tutoring effectively reduced discrepancies between symbolic and nonsymbolic number processing in children with MD, with their post-tutoring cross-format similarity in numerical processing aligned with the pre-tutoring levels of their TD peers.

### CFN tutoring leads to improvements in arithmetic fluency in children with MD

To further assess the effectiveness of CFN tutoring, we examined its impact on arithmetic fluency in children with MD. As the tutoring program specifically focused on enhancing children’s numerical understanding and did not include explicit training of arithmetic skills, children’s gains on arithmetic fluency served as an indicator of transfer of learning to broader math problem solving skills.

At baseline (pre-tutoring), arithmetic fluency in the MD group was significantly lower than that of the TD group (*t*(51) = 8.47, *p* < 0.001, Cohen’s *d* = 2.33) (Fig. 2b). Following the tutoring program, the MD group significantly improved on arithmetic fluency (*t*(25) = 3.41, *p* = 0.002, Cohen’s *d* = 0.89). However, unlike behavioral normalization observed in cross-format similarity in numerical processing, children with MD continued to show lower arithmetic fluency at post-tutoring compared to TD children at pre-tutoring (*t*(51) = 5.00, *p* < 0.001, Cohen’s *d* = 1.37) (see Supplementary Results for results from ANOVA). These findings suggest that CFN tutoring reduced the gap in arithmetic fluency between the two groups of children, though the performance gap between groups remained following tutoring.

Notably, additional analysis revealed that tutoring-induced gains in arithmetic fluency were significantly related to reduction in between-format dissimilarity in children with MD (*r*(26) = −0.393, *p* = 0.048; see details in Supplementary Results), which suggests that individual differences in transfer of learning to arithmetic fluency were associated with the degree of changes in cross-format similarity in numerical processing following CFN tutoring.

### Neural normalization in cross-format NRS following CFN tutoring in children with MD

Our next objective was to test the neural normalization hypothesis that our CFN tutoring leads to normalization of atypical neural representational patterns in the MD group to the level observed in TD children prior to tutoring (Fig. 1c). To test this hypothesis, we first examined whether cross-format NRS (see Fig. 1b and Methods) are different between well-matched children with MD and TD children before tutoring (see Supplementary Table 2 for group control). We then focused on the identified regions to assess whether post-tutoring cross-format NRS in children with MD became comparable to pre-tutoring cross-format NRS of their TD peers.

**Fig. 3:**
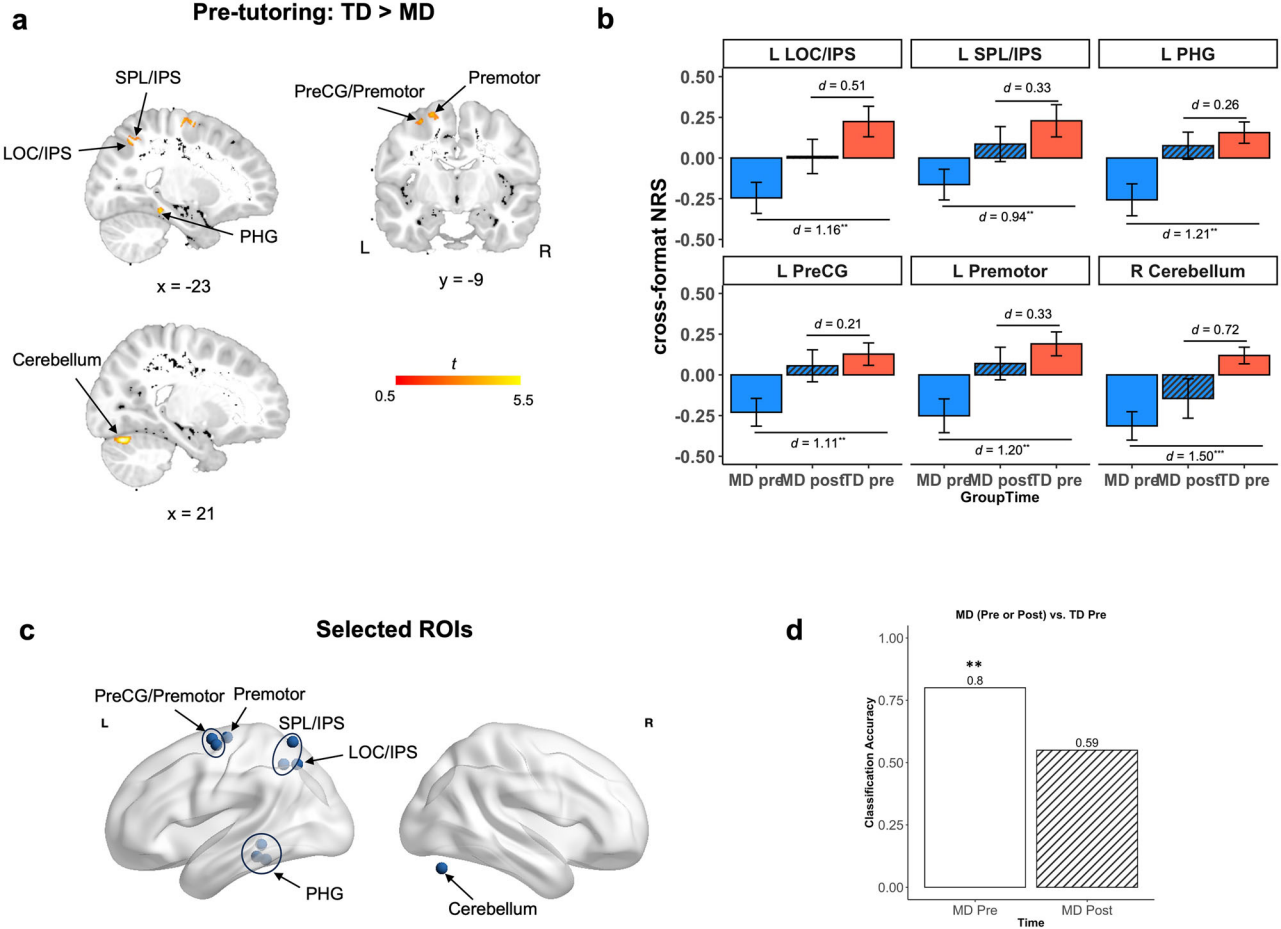
Neural normalization in children with MD following cross-format number tutoring. **a** Whole-brain analysis revealed pre-tutoring differences in cross-format NRS between the MD and TD groups in key brain regions implicated in memory and visuospatial numerical processing, including the PHG, SPL/IPS, LOC/IPS, PreCG, and Premotor. Figure represents the significant voxels overlaid onto the pediatric T1-weighted brain template. **b** Tutoring led to normalization of cross-format NRS in children with MD at post-tutoring, which was comparable to the pre-tutoring level in TD children (Supplementary Table 4). Effect sizes illustrate a reduction in neural disparity of the MD group following CFN tutoring, when compared to the TD group at pre-tutoring, across all brain regions. **c** Support-vector machine (SVM)-based classification was performed on 10 brain regions identified from the whole-brain analysis where children with MD exhibited lower pre-tutoring cross-format NRS, compared to their TD peers (Fig. 3a; see Supplementary Table 3 for details). **d** SVM classifier revealed significant difference in cross-format NRS between the MD and TD groups at pre-tutoring (*p* = 0.004), but not between the MD group at post-tutoring and the TD group at pre-tutoring (*p* = 0.232). ^**^p < 0.01, ^***^p < 0.001. *d* = Cohen’s *d*. MD = children with mathematical difficulties, NRS = neural representational similarity, TD = typically developing children, L = Left, R = Right.

At baseline (pre-tutoring), a whole-brain two-sample *t*-test revealed lower cross-format NRS in the MD group compared to the TD group in multiple distributed brain regions including the left intraparietal sulcus (IPS)^67,68^, parahippocampal gyrus (PHG), precentral gyrus (PreCG), premotor cortex, and right cerebellum (*p* < 0.005, cluster size = 547) (Fig. 3a, Supplementary Table 3). Notably, no brain region showed higher cross-format NRS in the MD group, compared to the TD group, which indicates that neural representations of symbolic and nonsymbolic numbers were less similar in the MD group before tutoring.

Crucially, increases in cross-format NRS were observed across these regions in children with MD at post-tutoring in comparison to pre-tutoring. Among the regions that the MD group had lower cross-format NRS than the TD group before tutoring, we found that CFN tutoring led to increases in cross-format NRS in the MD group in all ROIs, reaching levels comparable to the TD group at pre-tutoring (*FDR-corrected p*s > 0.290; Fig. 3b, Supplementary Table 4 and Supplementary Table 5).

Further validation of the neural normalization hypothesis came from multivariate classification analyses of aberrant NRS in the MD group across distributed brain regions. Linear support vector machine (SVM) was used for multivariate classification with cross-format NRS from 10 brain regions identified from the whole-brain analysis before tutoring as input (see Methods, Supplementary Table 3, Fig. 3c). SVM significantly differentiated MD and TD groups at pre-tutoring (accuracy = 0.80, *p* = 0.004; Fig. 3d) as expected, but failed to differentiate cross-format NRS in the MD group at post-tutoring from that of TD group at pre-tutoring (accuracy = 0.59, *p* = 0.232) (Fig. 3d).

### Cross-format association and dissociation of numbers following CFN tutoring

Our final objective was to test the model of association and dissociation between number formats (Fig. 1d). To this aim, we characterized the patterns of behavioral and neural representational changes in children with MD (i.e., children who are in relatively earlier stages of math skill development) and their TD peers (i.e., children who are in relatively later stages of math skill development) following CFN tutoring. Specifically, we examined whether CFN tutoring induced similar or distinct patterns of changes in cross-format similarity in numerical processing (between-format dissimilarity) and NRS across the two groups.

To test cross-format similarity in numerical processing, we conducted a mixed-design ANOVA with Group (MD, TD) as the between-subject factor and Time (pre-tutoring, post-tutoring) as the within-subject factor. We found a significant interaction between Group and Time on between-format dissimilarity (*F*(1,51) = 10.17, *p* = 0.002, *η*^2^ = 0.08). Follow-up paired t-tests revealed that between-format dissimilarity decreased with training in the MD group (*t*(25) = −1.93, *p* = 0.064, Cohen’s *d* = −0.56), while it increased with training in the TD group (*t*(26) = 2.71, *p* = 0.012, Cohen’s *d* = 0.55). No other main effects were significant (*ps* > 0.815). (Supplementary Fig. 2). These findings indicate increased cross-format similarity in numerical processing in children with MD and decreased cross-format similarity in TD children.

**Fig. 4:**
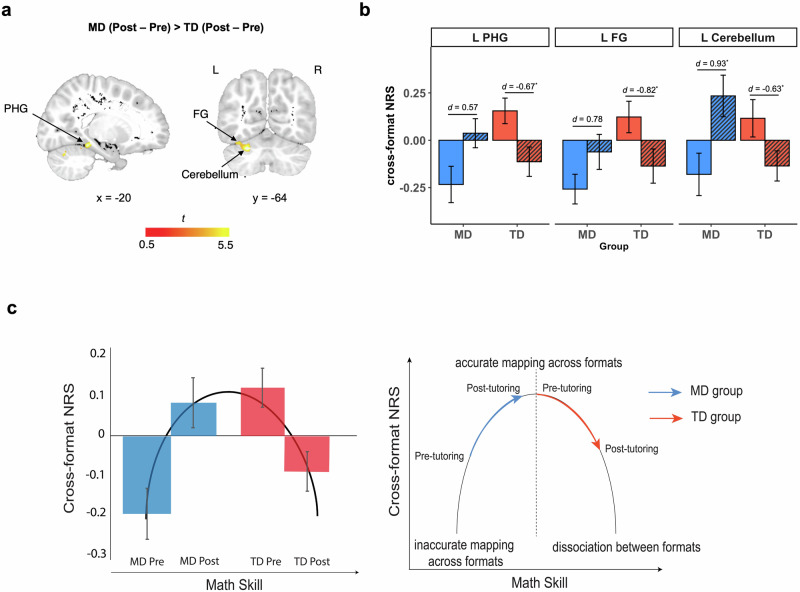
Differential neurodevelopmental trajectories of math skill development in the MD and TD groups following cross-format number tutoring. **a** Time (pre-tutoring, post-tutoring) x Group (MD, TD) ANOVA revealed a significant Time x Group interaction on cross-format NRS in 5 brain regions, including the parahippocampal gyrus (PHG), occipital fusiform gyrus (FG), and cerebellum. Figure represents the significant voxels overlaid onto the pediatric T1-weighted brain template. **b** Time x Group interaction effect was characterized by relative increases in cross-format NRS in the MD group and relative decreases in cross-format NRS in the TD group in observed brain regions. **c** The observed changes across the two groups aligned with distinct patterns of cross-format association and dissociation of numbers: on average, children with MD showed enhanced cross-format NRS at post-tutoring, reaching levels similar to pre-tutoring TD levels; TD children shifted towards more specialized, distinguishable neural representations between the two number formats, aligned with their increased proficiency in symbolic numbers. See Supplementary Table 6 and Supplementary Table 8 for details. ^*^p < 0.05. *d* = Cohen’s *d*. MD = children with mathematical difficulties, NRS = neural representational similarity, TD = typically developing children, L = Left, R = Right.

To test the profiles of neural plasticity in children with MD and their TD peers following CFN tutoring (Fig. 1d), we performed a whole-brain ANOVA on cross-format NRS with Group (MD, TD) and Time (pre-tutoring, post-tutoring) as between- and within-subject factors (see Methods). Here we observed a significant interaction between Group and Time in the left PHG, occipital fusiform gyrus (FG), and cerebellum, pointing to differential tutoring-induced neural representational changes in these brain regions between the two groups (see Fig. 4a, Supplementary Table 6). In addition to the interaction effect, only a main effect of Group was observed (see Supplementary Table 7) in regions similar to those observed in our group *t*-tests (see Supplementary Table 3). Importantly, the observed significant interaction between Group and Time from the whole-brain ANOVA indicated distinct tutoring-related changes in cross-format NRS between children with MD and TD children.

Follow-up regional-level analysis revealed that the significant interaction between Group and Time was characterized by a significant decrease in the TD group (*t*(19) = −2.36, *p* = 0.036, Cohen’s *d*s = −0.63) across several regions, including the left cerebellum, FG and PHG (*t*s(19) < −2.36, *p*s > 0.036, |Cohen’s *d*s | > 0.54), as well as a significant increase in cross-format NRS in the MD group (*t*(15) = 3.49, *p* = 0.015, Cohen’s *d*s = 0.93) in the left cerebellum (Fig. 4b, Supplementary Table 8 and Supplementary Table 9). These findings point to potentially divergent trajectories of neural plasticity between the two groups of children.

Next, we examined the overall profile of neurodevelopmental differences between groups in response to CFN tutoring, by averaging cross-format NRS across 5 brain regions (1 region in the PHG, 1 region in the FG, and 3 regions in cerebellum) that showed a significant Group by Time interaction in the whole-brain ANOVA (Fig. 4b, Supplementary Table 6). The significant interaction between Group and Time was maintained when using averaged cross-format NRS (*F*(1,68) = 19.38, *p* < 0.001, *η*^2^ = 0.22). These patterns of changes reflect distinct trajectories of association and dissociation between two number formats from earlier (MD group) to later (TD group) stages of math skill development (Fig. 4c).

To further validate these findings at a network level, we conducted a multivariate classification analysis using SVM with cross-format NRS data extracted from the 5 ROIs that exhibited significant Group x Time interaction effects in the whole-brain ANOVA as input (Supplementary Table 6). SVM accurately differentiated post- vs pre-tutoring differences in cross-format NRS (i.e., neural plasticity patterns) between MD and TD groups (accuracy = 0.725, *p* = 0.024). These results demonstrate distinct patterns of tutoring-induced plasticity in cross-format NRS between the two groups.

## Discussion

We implemented a 4-week, personalized cross-format number (CFN) tutoring program designed to enhance foundational numerical skills in children with mathematical difficulties (MD). Our CFN tutoring program targeted the integration of symbolic and nonsymbolic numbers in the earlier stages of tutoring, progressively transitioning to an emphasis on symbolic numerical processing in its later stages. We examined tutoring-induced changes in cross-format similarity between symbolic and nonsymbolic numbers in children with MD, in comparison to TD controls. Our neural representational similarity (NRS) analysis allowed us to examine if CFN tutoring could normalize cross-format NRS between symbolic and nonsymbolic numbers in children with MD, offering a more nuanced understanding than traditional univariate analyses^64–66^. By uncovering the neural mechanisms underlying enhancement of weak numerical skills in children with MD, our study provides essential insights into effective interventions that address the unique learning needs of these children.

We highlight three key findings, each aligned with specific hypotheses tested. First, weak cross-format similarity between symbolic and nonsymbolic number discrimination in children with MD was normalized following CFN tutoring. Remarkably, CFN tutoring also improved arithmetic fluency in children with MD, suggesting that the program could induce transfer of learning to broader math problem-solving skills in these children. Second, multivariate neural pattern analysis revealed normalization of cross-format NRS in children with MD. Post-tutoring, cross-format NRS in children with MD aligned with pre-tutoring level in TD controls, in line with our neural normalization hypothesis. Third, our analysis revealed that CFN tutoring induced distinct patterns of change in cross-format similarity in numerical processing and NRS in children with MD compared to their TD peers. These findings deepen our understanding of learning and neural plasticity in children with MD and have implications for the development of personalized intervention for children with neurodiverse abilities.

The first objective of our study was to determine the effectiveness of a 4-week CFN tutoring in remediating behavioral measure of cross-format numerical mapping in children with MD compared to TD children. Prior to tutoring, children with MD showed lower cross-format similarity between symbolic and nonsymbolic number comparison, compared to TD children. This result aligns with previous research suggesting that children with MD have less precise mapping and different processing ability between symbolic numbers and nonsymbolic quantities^14,25–29^. Remarkably, following tutoring, children with MD exhibited a level of cross-format similarity in numerical processing that is comparable to their TD peers prior to tutoring. Thus, our results suggest that the 4-week CFN tutoring program was effective in remediating low levels of similarity in processing across symbolic and nonsymbolic numbers in children with MD.

We further assessed transfer of learning to broader math problem-solving skills in children with MD by examining the impact of the CFN tutoring program on their arithmetic fluency. Notably, children with MD showed significant improvements on arithmetic fluency even though they were specifically trained on basic numerical fluency, such as counting and comparison, and were not instructed on arithmetic principles. Unlike other interventions that incorporate explicit instructions on arithmetic problem solving^51,52,69^, our program was specifically designed to bolster numerical fluency across different formats. This finding is particularly noteworthy given the scarcity of intervention studies focusing on symbolic and nonsymbolic cross-format numerical mapping without introducing arithmetic in children with MD. Our results suggest that targeted cross-format number training can contribute to broader gains in arithmetic problem-solving skills for children with MD even in the absence of explicit arithmetic training.

Additionally, our analysis revealed a significant relation between improvements in arithmetic fluency and changes in cross-format similarity in numerical processing in children with MD, indicating that enhanced integration between symbolic and nonsymbolic number formats may play a role in bolstering arithmetic skills that were not directly targeted by the tutoring program. This connection emphasizes the critical contribution of cross-format numerical understanding to broader mathematical competencies. Together, our findings highlight the effectiveness of cross-format number training in not only promoting learning, but also meaningful transfer to arithmetic problem-solving, specifically in children with MD.

The second objective of our study was to test the neural normalization hypothesis by examining whether aberrant levels of cross-format NRS in children with MD can be normalized following CFN tutoring. At pre-tutoring baseline, the results revealed lower cross-format NRS in the MD group compared to the TD group in distributed brain areas, including the intraparietal sulcus, parahippocampal gyrus, premotor cortex, and cerebellum. Crucially, after CFN tutoring, levels of cross-format NRS in the MD cohort across all these brain regions became comparable to those of TD children at their baseline. Furthermore, multivariate classification analysis revealed a lack of distinction between post-tutoring cross-format NRS in children with MD and pre-tutoring cross-format NRS in TD children. These findings provide converging evidence that the CFN tutoring program facilitated neural representational plasticity in children with MD by strengthening neural mapping between symbolic and nonsymbolic numbers to baseline levels observed in their TD counterparts.

Brain regions exhibiting atypical cross-format NRS patterns in children with MD prior to tutoring are recognized as crucial for numerical cognition^70–74^. The posterior parahippocampal gyrus within the medial temporal lobe is crucial for encoding spatial memories into cohesive cognitive structures^75–79^, a process likely essential for forming associations nonsymbolic number with symbolic number formats^72^. The intraparietal sulcus is associated with representation and manipulation of numerical quantity as well as spatial attention^67,70,72,80–83^, and has been consistently identified as a key region of deficit in children with mathematical learning difficulties^29,71,84–86^. The dorsal preCG and premotor area are frequently activated during numerical judgement tasks in children^87,88^, possibly reflecting finger-counting habits^89^. Lastly, lesion studies suggest that the cerebellum is critical for iterative procedures involved in calculations^90^, and children with mathematical difficulties exhibit both functional^84,91^ and structural^92^ cerebellar deficits. It is noteworthy that CFN tutoring led to normalization of aberrant cross-format NRS in children with MD in all these brain regions, bringing them on par with pre-tutoring levels observed in TD children. These findings are consistent with previous observations of normalization of widespread aberrations in brain activation^84^ and connectivity^52^ in children with MD following math interventions involving arithmetic problem solving.

Taken together, our findings indicate that the neural normalization of cross-format number representations in children with MD involves a network of distributed brain areas, aligns with systems neuroscience models of neural systems and pathways that are implicated in impairments underlying MD^71,85,93^. This network-level neural normalization in children with MD demonstrates the efficacy of CFN tutoring and emphasizes its potential in remediating atypical neural representations across different numerical formats in affected children.

The third objective of our study aimed to determine whether the CFN tutoring program differentially influences behavioral and neural plasticity in children with MD and TD children. Our analysis revealed significant Time x Group interactions for both behavioral and neural measures of cross-format numerical mapping, pointing to diverging trajectories of changes in cross-format numerical processing and neural representational plasticity following tutoring.

Behaviorally, children with MD showed a marked increase in cross-format similarity in processing across symbolic and nonsymbolic numbers, while TD children, in contrast, showed a decrease in this cross-format similarity after tutoring. In line with our hypothesis, these contrasting behavioral profiles suggest that CFN tutoring focused on integration of symbolic and nonsymbolic number representations exerts distinct patterns of behavioral changes in cross-format numerical mapping depending on children’s initial math ability.

At the neural level, our NRS analysis revealed distinct patterns of tutoring-related changes in basal ganglia regions which are pivotal for skill acquisition^94,95^. Specifically, children with MD, who initially showed lower cross-format NRS indicative of suboptimal neural mapping, exhibited an increase in cross-format NRS post-tutoring. This enhancement suggests improved neural mapping as a result of tutoring. In contrast, TD children, who initially displayed higher cross-format NRS prior to tutoring, demonstrated a decrease post-tutoring, indicating a shift towards greater dissociation between symbolic and nonsymbolic numerical processing.

These observations address the two competing hypotheses proposed in our study. Initially, we posited a linear model consistent with the ‘symbolic estrangement’ theory, which predicts a progressive dissociation of numerical formats with training. However, our findings support a U-shaped model, characterized by decreases in NRS among TD children, contrasting with increases in children with MD. This nonlinear profile of neural plasticity points to potentially distinct learning pathways in numerical cognition between children with MD and their TD peers.

Our results not only corroborate our proposed model of cross-format association and dissociation across varying levels of mathematical skills but also extend the theory of ‘symbolic estrangement’^35,37,96^. Our study provides new insights into the varied impact of CFN tutoring depending on the stage of math skills, as demonstrated by the divergent patterns of behavioral and neural plasticity between children with MD and TD children. These findings underscore the importance of accounting for initial math ability when aiming to mitigate learning disparities between children with MD and their TD peers and optimizing learning for all children.

One limitation of our study is the modest sample size and absence of a no-contact control group, constraints necessitated by the extensive resources required to conduct a rigorous intervention and collect high-quality behavioral and neuroimaging data from well-matched groups of children with MD and TD peers. Additionally, mathematics instruction at school or home may have influenced observed learning improvements. Importantly, our primary goal was to directly compare the effects of tutoring between children with MD and their TD peers; future studies should incorporate larger samples and control conditions to further delineate specific effects of targeted tutoring. Longitudinal studies are needed to fully understand the onset and evolution of the nonlinear inverted U profile observed in our study. Such studies are crucial to determine whether additional training might lead to decreases in NRS, suggesting a progression towards pattern separation and ‘symbolic estrangement’ akin to that observed in TD children. This research could reveal whether children with MD eventually exhibit a dissociation between symbolic and nonsymbolic representations of quantity—indicative of a developmental delay^97^, face persistent challenges in achieving stable neural dissimilarity between number formats^98^, or demonstrate significant variability in their learning profiles and outcomes^99^. These longitudinal insights are essential for developing interventions that address immediate learning deficits while also promoting sustained academic success and cognitive development in children with MD.

In conclusion, our innovative cross-format number (CFN) tutoring program significantly enhanced numerical and arithmetic skills in children with mathematical difficulties (MD), demonstrating behavioral improvements across various mathematical tasks. These enhancements suggest that even short-term interventions can have substantial effects on children’s ability to link nonsymbolic (sets of objects) to symbolic (Arabic numerals) number representations, providing a robust foundation for their ongoing mathematical development. Our study also elucidated the neural mechanisms by which a short-term, cross-format number tutoring program ameliorates weak numerical abilities in children with MD. By employing multivariate neural pattern analysis, we advanced beyond traditional univariate methods to illuminate how the brain organizes and differentiates numerical formats, and how tutoring influences neural plasticity in these children. Significantly, CFN tutoring normalized aberrant neural representational patterns in children with MD, indicating extensive, tutoring-induced functional reorganization within critical brain regions associated with numerical cognition.

Furthermore, our findings highlight distinct patterns of neural plasticity in children with MD compared to their TD peers, enhancing our understanding of their unique learning trajectories. These results are vital for developing targeted, evidence-based interventions aimed at closing the performance gap between children with MD and their TD peers, ensuring that interventions are not only effective in the short term but also contribute to long-term educational outcomes.

## Methods

### Participants

The sample for the behavioral analyses included 53 children (27 female; 25 children with MD and 28 TD; mean age = 8.16 years, SD = 0.66). For the fMRI analyses, we applied stricter inclusion criteria related to head motion and image quality (see details in fMRI data analysis section below), resulting in a final sample of 36 children (19 female; 16 with MD and 20 TD; mean age = 8.20 years, SD = 0.60). All participants were second- and third-grade elementary school students recruited from multiple school districts as part of a larger longitudinal investigation of numerical cognition^39^. Reasons for excluding participants from the fMRI analysis included excessive head motion during scanning (*n* = 13), poor image quality (*n* = 3), and inadequate co-registration to the standardized brain template (*n* = 1). These excluded participants were retained in behavioral analyses, as behavioral data quality was not impacted by these imaging factors.

The research has been conducted in accordance with the Declaration of Helsinki and institutional ethical guidelines. All protocols were approved by the Institutional Review Board at Stanford University (11849: Longitudinal Brain Imaging Studies of Cognitive Functions). Informed written consent was obtained from the child’s legal guardian, as all participants were under 16 years of age.

### Definition of MD and TD groups

MD and TD groups were defined using criterion-based cutoff scores from math tests similar to previous studies investigating children with MD^84,100–104^. Children with MD scored at or below 90 (i.e., the 25th percentile) and TD children scored above 90 on Math Fluency subtest of the Woodcock Johnson-III (WJ-III) (Woodcock et al. 2001). We acknowledge that we do not make a distinction between children with persistent developmental dyscalculia and those with milder forms of math difficulties as we did not test if these children had math difficulties that persisted for at least 6 months. Importantly, we identified children with MD and TD children based on specific performance differences in math scores and comparable scores on other domain-general cognitive measures (see Supplementary Table 2).

### Symbolic and nonsymbolic number comparison tasks

The present study aimed to examine children’s cognitive and brain plasticity in response to CFN tutoring. Children underwent brain imaging sessions and cognitive assessments before and after a 4-week CFN tutoring. During brain imaging sessions, children completed symbolic and nonsymbolic number comparison tasks in the scanner wherein they determined the larger between two nonsymbolic (dot arrays) or symbolic (Arabic numerals) numbers presented on each side of the screen (Fig. 1b). A total of 64 trials was presented in each run. Numbers between 1 and 9, excluding 5, were presented. We used a 2 × 2 experimental design accounting for both the size of the number pair (little, big) and the distance between the numbers of the pair (near, far), resulting in 16 trials per condition. In half of the trials, the sum of the pair was greater than 10 (“big” numbers), and in the other half, it was less than 10 (“little” numbers). In half of the trials, the distance between the numbers was 1 unit (“near” distance), and in the other half, the distance was 5 units (“far” distance).

On each trial, a fixation appeared for 500 ms followed by a pair of quantities which remained visible for 1000 ms and a blank screen for 1500 ms to fill up the response phase. Stimuli were presented for short duration to avoid counting of dots in the nonsymbolic condition. For the nonsymbolic number comparison task, (i) the total area covered by each array of dots and (ii) the average size of dots in each array were controlled to account for potential confounds with the number of items. Each pair of quantities was presented 4 times, twice with the larger number on the left, and twice with the larger number on the right.

Using a button box (2-button fiber optic response pad, Curdes system: www.Curdes.com), children indicated which quantity was larger by pressing the left button if the larger number was on the left side or the right button if the larger number was on the right side. Stimuli were presented using E-Prime and displayed using an LCD projector and a back-projection screen in the scanner suite. The inter-trial interval between trials was jittered randomly between 1.7 and 3.8 s. Total run duration was ~6 min. To evaluate the effectiveness of the cross-format number intervention, we primarily focused on between-format dissimilarity, calculated from the performance on symbolic and nonsymbolic number comparison tasks. Details of this measure are further explained in the Behavioral analysis section.

### Cognitive assessments

In addition to symbolic and nonsymbolic number comparison tasks, children completed a battery of cognitive assessments to assess their arithmetic abilities as well as IQ, reading abilities, and working memory, described below in details. Demographics and scores from cognitive assessments at the time of inclusion are shown in Supplementary Table 2.

To measure intelligence quotient (IQ) scores, the Wechsler Abbreviated Scale of Intelligence™ (WASI; Wechsler, 1999), was administered to measure Verbal IQ (VIQ), Performance IQ (PIQ), and Full-Scale IQ (FSIQ). Reading abilities were assessed using Letter Word Identification and Word Attack subtests of the WJ-III (Woodcock et al. 2001). In the Letter Word Identification subtest, children were asked to fluently read letters and words of increasing difficulty. In the Word Attack subtest, children were asked to read pseudo words of increasing complexity. To assess working memory, eight subtests of Automated Working Memory Assessments (AWMA) were used. Digit Recall and Word Recall subtests assessed verbal short-term memory. Backward Digit Recall subtest assessed verbal working memory. Block Recall subtest assessed visuo-spatial short-term memory. Spatial Recall subtest assessed visuo-spatial working memory. Lastly, children’s arithmetic ability was assessed from Math Fluency subtest of the WJ-III^105^ to investigate potential transfer of learning. This subtest is a timed pencil and paper test that measures individual’s ability to quickly and accurately solve simple addition, subtraction and multiplication problems (Fig. 1a). Children were given a 3 min time limit and instructed to solve as many problems as they can. All problems were presented vertically and involved operands from 0 to 10. Given that the intervention did not directly address arithmetic skills, improvements in this area were viewed as indicative of the transfer of learning from the cross-format number training.

### CFN tutoring

Across four weeks, children underwent twelve sessions (3 sessions/week) of one-on-one tutoring specifically designed to enhance fundamental understanding of relations between symbolic and nonsymbolic numbers, as well as each number format processing. Quantities ranged from 1 through 9 to facilitate exact processing of numbers. Learning activities for children progressed gradually each week, aiming to build proficiency in exact symbolic number processing: children learned and practiced basic counting principles in *week 1*, processing of nonsymblic quantities in *week 2*, understanding relations between symbolic and nonsymbolic quantities in *week 3*, and lastly, processing of exact symbolic numbers in *week 4*. In each session, a trained tutor used various interactive learning tools including physical manipulatives and computer games. At the end of each session (except for first two sessions), children completed a review worksheet, which included a list of problems based on the week’s focus. Children received stickers upon completion of activities. Details are below.

In Week 1, children were introduced to the counting principle through lessons and a video clip demonstrating accurate or inaccurate counting of sock puppets. Children also played Restaurant Game^106^ in which they counted the number of dishes to cook for animals presented in the screen. At the end of Week 1, children completed a review worksheet, in which they counted the number of animals on the worksheet. After completion of Week 1 sessions, children were asked to verbally count from 1 to 9 before beginning sessions in Weeks 2−4.

In Week 2, children were introduced to comparison of nonsymbolic numbers using sets of erasers. Children played Math Circles wherein they determined a Math Circle with more erasers between two circles presented on the table. This lesson from week 2 was designed to increase children’s familiarity with number comparison. Starting from week 2, two interactive games with a tutor were introduced to children: 1) Math War^84^ in which children compared which of two quantities is larger and 2) Comparing speed in which children determined the quantity of one value above or below the given quantity. In the Math War, the child and the tutor had a deck of card with nonsymbolic numbers for each, and both flipped the card one at a time. The child wrote down the number on his/her own card and the one on the tutor’s card, and then determined the card with larger number. In the Comparing speed, the tutor laid four cards with nonsymbolic quantities from 4 to 7 on the table and then the child and tutor each took five cards from their own deck of cards. Next, they placed the card from their own deck on the top of the card on the table if the quantity on the card from their deck was below or above the quantity of the card on the table. When the child placed all the cards from their own deck, the game was completed. Lastly, children completed a review worksheet for Week 2, in which they determined which of the two nonsymbolic numbers was larger.

In Week 3, children were introduced to integration of symbolic and nonsymbolic representations of numbers through similar tutoring activities as Week 2. In the Math Circles, children compared a card presenting a set of erasers in a Math Circle to a card presenting a symbolic number (Arabic numeral). In the Math War and Comparing speed, quantities on the cards were in nonsymbolic or symbolic formats. For symbolic format, the child drew a number of dots that corresponds to the symbolic number on the card. Lastly, children completed a review worksheet for Week 3, in which they determined a larger quantity between symbolic and nonsymbolic numbers.

In Week 4, children practiced comparison between symbolic numbers through similar tutoring activities as Weeks 2 and 3. Children were introduced to an adapted version of Beat Your Score^63^ wherein they placed four decks of cards in numerical order for three times (the tutor shuffled the cards for each trial) with an aim of “beating” the time taken for the previous trial. Quantities on each deck of cards were in nonsymbolic (a dot array), mixed (a dot array and numerals), or symbolic formats. In Math War and Comparing speed, quantities on the cards were in symbolic format. Lastly, children completed a review worksheet for Week 4, in which they determined which of the two symbolic numbers was larger. Additional information on the tutoring protocol can be found in Park et al. (2024).

### Behavioral analysis

For *the symbolic and nonsymbolic number comparison* tasks, trials with response times below 150 ms—indicative of anticipatory responses—were excluded from analysis in accordance with established practices and field recommendations^107,108^. Prior studies on reaction time suggest that valid responses requiring stimulus encoding and decision-making do not typically occur before 100–200 ms^109–111^, with 150 ms generally considered a lower bound for meaningful cognitive processing^112,113^. Applying this criterion led to the exclusion of 2.86% of trials from the nonsymbolic task and 2.67% of trials from the symbolic task.

We computed children’s efficiency scores by dividing accuracy by median reaction times (RT) for each task to account for potential speed-accuracy trade-off^114^. We chose this formulation for two key reasons: (1) it provides a more intuitive interpretation, where higher scores indicate better performance (higher accuracy achieved in less time)^115^, and (2) it maintains consistency with previous developmental studies in this area^116,117^. Dissimilarity between symbolic and nonsymbolic number processing (between-format dissimilarity) was assessed by calculating absolute difference in efficiency between two comparison tasks. For this analysis, two children’s data were excluded due to poor performance in one (symbolic or nonsymbolic) comparison task.

To test *behavioral normalization hypothesis*, planned two-sample *t*-tests were conducted to assess (i) whether children with MD exhibit higher between-format dissimilarity than TD children before tutoring and (ii) whether between-format dissimilarity in children with MD after tutoring reach the level of TD children before tutoring. To further understand whether tutoring induced similar or different patterns of changes in between-format dissimilarity in number comparison ability between children with MD and TD children, we performed a mixed ANOVA with Group (MD, TD) as the between-subject factor and Time (pre-, post-tutoring) as the within-subject factor on between-format dissimilarity. Follow up two-sample and paired *t*-tests clarified significant effects from ANOVA.

As part of behavioral normalization hypothesis, planned two-sample *t*-tests were conducted to assess (i) group difference in arithmetic fluency (measured by WJ-III Math Fluency) before tutoring and (ii) difference in arithmetic fluency between MD group after tutoring and TD group before tutoring. To further understand tutoring-induced changes in arithmetic fluency in the two groups of children, we performed a mixed ANOVA with Group (MD, TD) as the between-subject factor and Time (pre-, post-tutoring) as the within-subject factor on arithmetic fluency. Follow up two-sample and paired t-tests clarified significant effects from ANOVA.

To further examine whether behavioral mapping between symbolic and nonsymbolic number formats may contribute to transfer of learning to arithmetic fluency following CFN tutoring, as observed in children with MD, we assessed correlation between changes in between-format dissimilarity (difference in task performance between two number formats) and gains in arithmetic fluency in these children. We additionally tested whether change in arithmetic fluency is related to improved format-specific task performance in each specific format (nonsymbolic or symbolic) in children with MD. Efficiency score was used to assess task performance.

To report effect size for all behavioral analyses, we used a generalized eta squared (*h*^2^) for ANOVA^118^ and Cohen’s *d* for *t*-tests^119^. Absolute Cohen’s d values of 0.2, 0.5, and 0.8 indicate small, medium, and large effect sizes, respectively.

### fMRI data acquisition and preprocessing

Functional images were acquired on a 3 T GE scanner (General Electric, Milwaukee, WI) using an 8-channel GE head coil. A T1-weighted, 132 slice high-resolution structural image was acquired at both pre- and post-tutoring scan sessions to facilitate registering each participant’s data to standard space. Head movement was minimized using additional pads and pillows around children’s head. A T2*-sensitive gradient echo spiral in-out pulse sequence^120^ was acquired with the following parameters: repetition time (TR) = 2000 ms, echo time (TE) = 30 ms, flip angle = 80°, field of view (FOV) = 220 mm, matrix size = 64 × 64, resolution = 3.44 × 3.44 × 4.5 mm^3^, interleaved. A total of 31 axial slices were acquired, 4 mm in thickness and 0.5 mm in spacing, covering the whole brain.

Images were preprocessed and analyzed using SPM12 (https://www.fil.ion.ucl.ac.uk/spm/). The first five volumes of each time-series were discarded to allow for signal equilibration. The preprocessing pipeline included realignment, slice-timing correction, co-registration to each subject’s T1-weighted anatomical image, spatial normalization to an age-appropriate pediatric Montreal Neurological Institute (MNI) brain template, resampling to 1 mm isotropic resolution using 4^th^ degree B-spline interpolation, and spatial smoothing with a 6 mm full-width at half-maximum (FWHM) Gaussian kernel to reduce spatial noise. Crucially, we employed a publicly available, age-appropriate pediatric MNI template from the NIHPD dataset constructed from MRI scans of 112 children aged 7 to 11 years^121^. This allowed us to better account for developmental anatomical variability and ensure optimal alignment with our participants.

Translational (x, y, z) and rotational (pitch, roll, yaw) motion parameters were estimated during the realignment stage of preprocessing. To ensure high data quality, only participants who completed both pre- and post-tutoring scans and met the following motion criteria were included in the final fMRI analysis: (1) total displacement in any direction not exceeding 2 voxels (6.88 mm) during either scan session; and (2) mean framewise displacement (scan-to-scan) not exceeding 0.5 mm, consistent with established thresholds for pediatric neuroimaging studies^122^. Applying these criteria resulted in the exclusion of four participants (3 with MD, 1 TD) from the original sample. These motion parameters did not significantly differ across tasks (nonsymbolic vs. symbolic), time points (pre- vs. post-tutoring), and groups (MD vs. TD) (see details in Supplementary Results).

### fMRI data analysis: First-level statistical analysis

Task-related brain activation was assessed using the general linear model (GLM) implemented in SPM12. In the first-level analysis, brain responses to each condition (i.e., little near, little far, big near, big far) were modeled using boxcar functions of 2500 ms, corresponding to the duration of a trial, convolved with a canonical hemodynamic response function and a temporal derivative to accommodate for voxel-wise latency differences in hemodynamic responses. Additionally, an error regressor was also included to explicitly account for and mitigate the influence of incorrect trials on our findings. To account for residual motion effects, all six head motion parameters (3 transitional and 3 rotational) were included as nuisance regressors in the first-level GLM for all participants. Serial correlations were accounted for by modeling the fMRI time-series as a first-degree autoregressive process. The GLM was applied to symbolic and nonsymbolic number comparison tasks separately. Voxel-wise contrast maps were generated for each participant for each task. The contrast of interest was the Near vs Far, which corresponds to the neural distance effect, to assess neural representations of quantity while controlling for low-level stimulus features and response demands.

### fMRI data analysis: Neural Representational Similarity (NRS)

To assess similarity in neural representation of quantity between formats, spatial correlation of multivariate patterns of brain activity between symbolic and nonsymbolic number comparison tasks was computed across the whole brain for each individual and each session. In contrast to measuring brain activation levels, NRS analysis provides a way to assess whether cognitive processes share similar neural features and to determine which brain areas are most sensitive to overlapping neural representation across symbolic and nonsymbolic number comparisons^123,124^. The NRS approach is based on well-grounded theories and findings on population coding and distributed representations^55^ and is ideal for examining underlying representations of mental states or cognitive functions.

Using a searchlight mapping method^125^, we obtained cross-format NRS of Near vs Far contrast across symbolic and nonsymbolic formats in the neighborhood surrounding each voxel of each individual’s brain. Specifically, a 6-mm spherical region centered on each voxel was selected, and cross-format similarity was computed within the sphere using the spatial correlation of voxel-wise brain activation (beta-weights). Searchlight maps were then created for every individual by going through every voxel across the whole brain. These searchlight maps were subsequently used for second-level analyses.

A few sets of second-level analyses were then conducted. First, to test the *neural normalization hypothesis*, we performed a whole-brain two-sample *t*-test contrasting MD and TD groups at pre-tutoring to identify the regions where the MD group shows atypical cross-format NRS levels compared to those of TD peers. Cross-format NRS of identified regions was compared between the MD group at post-tutoring and the TD group at pre-tutoring. Next, to test the *model of cross-format association and dissociation of numbers*, we performed a whole-brain mixed ANOVA with Group (MD, TD) as a between-subject factor, Time (Pre, Post) as a within-subject factor. The presence of a significant interaction between time and group would indicate a nonlinear pattern in brain plasticity between the groups, whereas the absence of such an interaction would indicate a linear pattern (Fig. 1d, e).

All statistical maps were masked with a grey matter mask, and significant clusters were identified using a height threshold of *p* < 0.005, similar to current practices in the fields of developmental cognitive neuroscience^35,126–128^, with whole-brain family-wise error rate correction at *p* < 0.05 (spatial extent of 574 voxels). The spatial extent threshold was determined through Monte Carlo simulations conducted on the pediatric grey matter mask with 5000 iterations. Follow-up regional-level analysis was based on estimated cross-format NRS in the regions identified from whole-brain analysis. False discovery rate (FDR) correction was used to correct for multiple comparisons in regional-level analysis. To confirm the robustness of our findings, we performed additional regional-level analysis using the full sample from Park et al. (2024) (19 children with MD and 21 TD children).

### fMRI data analysis: Multivariate classification analysis

To further confirm our results, we employed multivariate classification analysis using cross-format NRS values. Our classification analysis allowed us to test the neural normalization hypothesis and nonlinear neurodevelopmental model of cross-format association and dissociation of numbers at a larger scale across multiple brain regions.

First, to test neural normalization hypothesis, we used 10 regions of interest (ROIs) identified from the whole-brain two-sample *t*-test contrasting MD and TD groups at pre-tutoring in distributed brain regions, including the superior parietal lobule and intraparietal sulcus (SPL/IPS), lateral occipital cortex (LOC), precentral gyrus (preCG), premotor cortex, parahippocampal gyrus (PHG) and cerebellum (see details in Supplementary Table 3, Fig. 3c). We investigated whether cross-format NRS in the brain regions showing deficits in the MD group compared to their TD peers at pre-tutoring could be normalized post-tutoring, such that cross-format NRS in the MD group at post-tutoring is non-distinguishable from that of TD group at pre-tutoring. Thus, we performed a classification analysis to distinguish MD group at pre- or post-tutoring from TD group at pre-tutoring.

Second, to test the nonlinear neurodevelopmental model of association of number formats, we used 5 ROIs identified from the whole-brain ANOVA interaction between Time (pre, post) and Group (MD, TD) analysis, which included the left PHG, fusiform gyrus (FG), and cerebellum (see details in Supplementary Table 6, Fig. 4a). We investigated whether MD and TD groups show distinct patterns of changes in response to tutoring by performing a classification analysis to examine whether the MD group was distinguishable from the TD group based on differences in cross-format NRS between pre- and post-tutoring (Post – Pre). Thus, we performed a classification analysis to confirm whether cross-format NRS showed the patterns of neural changes in response to training were significantly different between MD and TD groups.

For all classification analyses, a linear support vector machine (SVM) classification algorithm with 10-fold cross-validation was used to assess the discriminability of cross-format NRS across each ROI set between groups. The python scikit-learn package (https://scikit-learn.org/) was used to perform this analysis. Permutation test (*n* = 5000) was used to assess the significance of classification accuracy.

## Supplementary information

Supplementary Information

## Data Availability

De-identified behavioral and fMRI data, consisting of individual-level statistical maps, will be available on the Open Science Framework (https://osf.io/29jue).

## Code availability

We employed R Version 4.3.2, SPM12, and Matlab Version R2022b for data analysis. Scripts used for the current study will be available on GitHub (https://github.com/scsnl/Park_npjSoL_2025).
