# Supplementary material for: Distinct neural representational changes following cross-format number tutoring in children with mathematical difficulties
Source: NPJ Sci Learn. 2025 Aug 13;10:52. doi: 10.1038/s41539-025-00345-y (PMC12350621; doi:10.1038/s41539-025-00345-y)
Supplement: Supplementary file 1 — Supplementary Information [file 41539_2025_345_MOESM1_ESM.pdf]

Supporting Information

Distinct neural representational changes following cross-format number tutoring in children with mathematical difficulties

Table of contents

I. **Supplementary Results**…..…..…..…..…..…..…..…..…..…..…..……..…… p. 2

II. **Supplementary Figures**…..…..…..…..…..…..…..…..…..…..…..……..…… p. 6

a. Supplementary Figure 1

b. Supplementary Figure 2

III. **Supplementary Tables**…..…..…..…..…..…..…..…..…..…..…..……………... p. 8

a. Supplementary Table 1

b. Supplementary Table 2

c. Supplementary Table 3

d. Supplementary Table 4

e. Supplementary Table 5

f. Supplementary Table 6

g. Supplementary Table 7

h. Supplementary Table 8

i. Supplementary Table 9

j. Supplementary Table 10

IV. **Supplementary References**…..…..…..…..…..…..…..…..…..……..…… p. 19

I. Supplementary Results

No significant effect of head motion

To confirm that observed differences in task (nonsymbolic, symbolic), time (pre-, post-tutoring), or group (MD, TD) were not confounded by head movement, we performed paired and two-sample *t*-tests on the seven motion parameters (3 translational and 3 rotational movements and mean scan-to-scan displacement). No significant differences in movement were found between nonsymbolic and symbolic number comparison tasks either before (*ps* > 0.356) or after (*ps* > 0.157) tutoring. Similarly, no significant differences between pre- and post-tutoring were observed within either task (nonsymbolic: *ps* > 0.051; symbolic: *ps* > 0.154). Furthermore, there were no significant differences in movement between MD and TD groups at either time point for either task (nonsymbolic at pre-tutoring: *ps* > 0.521; nonsymbolic at post-tutoring: *ps* > 0.317; symbolic at pre-tutoring: *ps* > 0.464; symbolic at post-tutoring: *ps* > 0.385).

Effects of tutoring on cross-format similarity in numerical processing assessed by accuracy and reaction time on number comparison tasks.

We examined whether CFN tutoring remediated weak cross-format similarity in numerical processing in children with MD to the level of TD children. As a measure of cross-format similarity in numerical processing, a metric of *between-format dissimilarity* was assessed by absolute difference in behavioral performance between nonsymbolic and symbolic number comparison tasks (|Nonsymbolic – Symbolic|). In addition to efficiency described in the main manuscript, we assessed between-format dissimilarity based on measures of accuracy and reaction time.

For between-format dissimilarity assessed by accuracy, two sample *t*-tests revealed no significant difference between the MD and TD groups at pre-tutoring ($p = 0.402$, Cohen's $d = 0.23$), nor between the MD group at post-tutoring and the TD group at pre-tutoring ($p = 0.928$, Cohen's $d = 0.03$) (**Supplementary Figure 1a**). A post-hoc paired *t*-test also indicated no significant tutoring-induced changes in the MD group ($p = 0.185$, Cohen's $d = 0.31$).

For between-format dissimilarity assessed by reaction time, we found similar patterns of results as that assessed by efficiency. Two sample *t*-tests revealed a significant difference between the MD and TD groups at pre-tutoring with moderate effect size ($t(51) = -2.19$, $p = 0.033$, Cohen's $d = -0.60$), and no significant difference between the MD group at post-tutoring and the TD group at pre-tutoring ($p = 0.503$, Cohen's $d = 0.19$) (**Supplementary Figure 1b**). A post-hoc paired *t*-test confirmed a significant decrease in between-format dissimilarity in the MD group following tutoring ($t(25) = -2.57$, $p = 0.016$, Cohen's $d = -0.73$). These results indicate that the observed normalization in efficiency-based between-format dissimilarity across children with and without MD were potentially driven by changes in reaction time, suggesting that CFN tutoring may have induced similar processing speed and efficiency between nonsymbolic and symbolic number comparison tasks in children with MD to levels of their TD peers.

Next, we conducted a mixed-design ANOVA with Group (MD, TD) as a between-subject factor and Time (pre-tutoring, post-tutoring) as a within-subject factor to examine whether CFN tutoring induced similar or distinct patterns of changes in between-format dissimilarity between the two groups of children.

For between-format dissimilarity assessed by accuracy, no significant main or interaction effects was observed ($ps > 0.270$, |Cohen's d| < 0.32) (**Supplementary Figure 2b**). For between-format dissimilarity assessed by reaction times, we found a significant Group by Time interaction effect ($F(1,51) = 7.22$, $p = 0.010$, $\eta^2 = 0.051$). Follow-up paired t-tests revealed a significant reduction in between-format dissimilarity in the MD group ($t(25) = -2.57$, $p = 0.016$, Cohen's $d = -0.73$), and no significant changes in the TD group after tutoring ($p = 0.433$, Cohen's $d = 0.12$) (**Supplementary Figure 2c**). No significant main effects of Group or Time were found ($ps > 0.548$).

Taken together, these results indicate distinct tutoring-induced changes in between-format dissimilarity in reaction time between the MD and TD groups and converge with the patterns of behavioral normalization in children with MD.

Increased in cross-format similarity in numerical processing was related with gains in arithmetic fluency in children with MD

We found that tutoring-induced gains in arithmetic fluency in children with MD were negatively correlated with changes in between-format dissimilarity ($r(26) = -0.393$, $p = 0.047$), but not with changes in format-specific task performance ($|rs| < 0.201$, $ps > 0.303$). These results indicate that a greater reduction in dissimilarity (or increased similarity) between nonsymbolic and symbolic numbers, rather than format-specific performance improvements, may have contributed to the observed transfer of learning to arithmetic fluency in children with MD.

Effects of tutoring on format-specific number comparison task performance

We examined whether CFN tutoring remediated number comparison ability in each format (nonsymbolic and symbolic) in children with MD. We assessed behavioral performance using efficiency, accuracy, and reaction time, as described below.

Efficiency. Two sample t-tests revealed no significant difference between children with MD and TD children at pre-tutoring (nonsymbolic: $p = 0.929$, Cohen's $d = -0.03$; symbolic: $p = 0.109$, Cohen's $d = -0.45$), nor between children with MD at post-tutoring and TD children at pre-tutoring (nonsymbolic: $p = 0.139$, Cohen's $d = 0.42$; symbolic: $p = 0.501$, Cohen's $d = 0.19$). These results suggest that children with MD did not show specific format-based impairments in number comparison ability before tutoring.

To further examine whether CFN tutoring induced similar or distinct patterns of changes in number comparison ability between the MD and TD groups, we conducted a mixed-design ANOVA on efficiency with Group (MD, TD) as a between-subject factor and Time (pre-

tutoring, post-tutoring) as a within-subject factor for each number format. For symbolic comparison, we found a significant main effect of Time ($F(1,51) = 38.02, p < 0.001, \eta^2 = 0.109$) and a marginally significant main effect of Group ($F(1,51) = 3.832, p = 0.051, \eta^2 = 0.061$) with medium effect size, but no significant interaction ($p = 0.833$). Similarly, for nonsymbolic comparison, we found a significant main effect of Time ($F(1,51) = 17.97, p < 0.001, \eta^2 = 0.073$), but no significant group or interaction effects ($p > 0.335$). Post-hoc paired *t*-tests confirmed significant improvements in efficiency from pre- to post-tutoring in both groups (MD: nonsymbolic: $t(25) = 2.16, p = 0.040$, Cohen's $d = 0.39$; symbolic: $t(25) = 3.72, p = 0.001$, Cohen's $d = 0.60$; TD: nonsymbolic: $t(26) = 3.94, p < 0.001$, Cohen's $d = 0.75$; symbolic: $t(26) = 5.25, p < 0.001$, Cohen's $d = 0.77$). These results suggest that CFN tutoring induced improvements on both symbolic and nonsymbolic comparison ability across the MD and TD groups.

Accuracy. Planned paired t-tests showed no significant improvements in the MD group for either task (nonsymbolic: $p = 0.083$, Cohen's $d = -0.37$; symbolic: $p = 0.151$, Cohen's $d = -0.28$). Two-sample t-tests also revealed no significant group differences at pre-tutoring (nonsymbolic: $p = 0.326$, Cohen's $d = -0.27$; symbolic: $p = 0.083$, Cohen's $d = 0.49$) or between the MD group at post-tutoring and the TD group at pre-tutoring (nonsymbolic: $p = 0.988$, Cohen's $d = -0.004$; symbolic: $p = 0.428$, Cohen's $d = -0.22$).

To further unpack the similar learning trajectories between the MD and TD groups identified through the efficiency measure in the main text, we conducted the same mixed-design ANOVA on accuracy with Group and Time factors for each comparison task. No significant main effects or interactions were observed in either task (nonsymbolic: $ps > 0.178$; symbolic: $ps > 0.159$). Post-hoc *t*-tests confirmed the absence of significant changes in accuracy in both groups (nonsymbolic: $ps > 0.082$, Cohen's $|d| < 0.37$; symbolic: $ps > 0.151$, Cohen's $|d| < 0.28$).

Reaction time. Planned paired t-tests showed significant reductions in reaction times in the MD group for both tasks (nonsymbolic: $t(25) = -2.45, p = 0.022$, Cohen's $d = -0.40$; symbolic: $t(25) = -3.56, p < 0.002$, Cohen's $d = -0.48$). However, two-sample t-tests revealed no significant group differences at pre-tutoring (nonsymbolic: $p = 0.426$, Cohen's $d = -0.22$; symbolic: $p = 0.148$, Cohen's $d = -0.40$) or between the MD group at post-tutoring and the TD group at pre-tutoring (nonsymbolic: $p = 0.408$, Cohen's $d = 0.23$; symbolic: $p = 0.602$, Cohen's $d = 0.14$).

To further unpack the similar learning trajectories between the MD and the TD groups identified through the efficiency measure in the main text, we conducted the same mixed-design ANOVA on reaction time with Group and Time factors for each comparison task. Here we found significant main effects of Time for both tasks (nonsymbolic: $F(1,51) = 18.34, p < .001, \eta^2 = 0.059$; symbolic: $F(1,51) = 22.21, p < 0.001, \eta^2 = 0.067$), but no significant effects of Group or Group x Time interactions ($ps > 0.129$). Post-hoc *t*-tests revealed significant pre- to post-tutoring reductions in reaction times across both groups and tasks (TD: nonsymbolic: $t(26) = -3.94, p < 0.001$, Cohen's $d = -0.62$; symbolic: $t(26) = -3.07, p = 0.005$, Cohen's $d = -0.53$; MD: nonsymbolic: $t(25) = -2.45, p = 0.022$, Cohen's $d = -0.40$; symbolic: $t(25) = -3.56, p < 0.002$, Cohen's $d = -0.48$).

Taken together, these findings suggest that tutoring-induced changes in efficiency were mainly driven by changes in reaction times in the MD group, and confirmed that the MD group did not exhibit deficits in performing number comparison tasks. Additionally, these results indicate that CFN tutoring led to comparable levels of improvement across both the MD and TD groups.

CFN tutoring leads to distinctive pattern of changes in arithmetic fluency in children with and without MD

To further examine whether CFN tutoring induced similar or distinct patterns of changes in arithmetic fluency between the MD and TD groups, we conducted a mixed-design ANOVA with Group (MD, TD) as a between-subject factor and Time (pre-tutoring, post-tutoring) as a within-subject factor. We found a significant main effect of Group ($F(1,51) = 43.51$, $p < 0.001$, $\eta^2 = 0.39$) and a significant interaction between Group and Time ($F(1,51) = 7.537$, $p = 0.008$, $\eta^2 = 0.04$). The main effect of Time was not significant ($p = .121$). Post-hoc paired t-tests revealed a significant improvement in arithmetic fluency in the MD group ($t(25) = 3.41$, $p = 0.002$, Cohen's $d = 0.89$), while no significant change was observed in the TD group ($p = 0.453$, Cohen's $d = 0.13$).

These results suggest that CFN tutoring, which focused on integration of nonsymbolic and symbolic number representations, induced distinct patterns of changes in arithmetic fluency across groups, with significant gains observed in children with MD.

II. Supplementary Figures

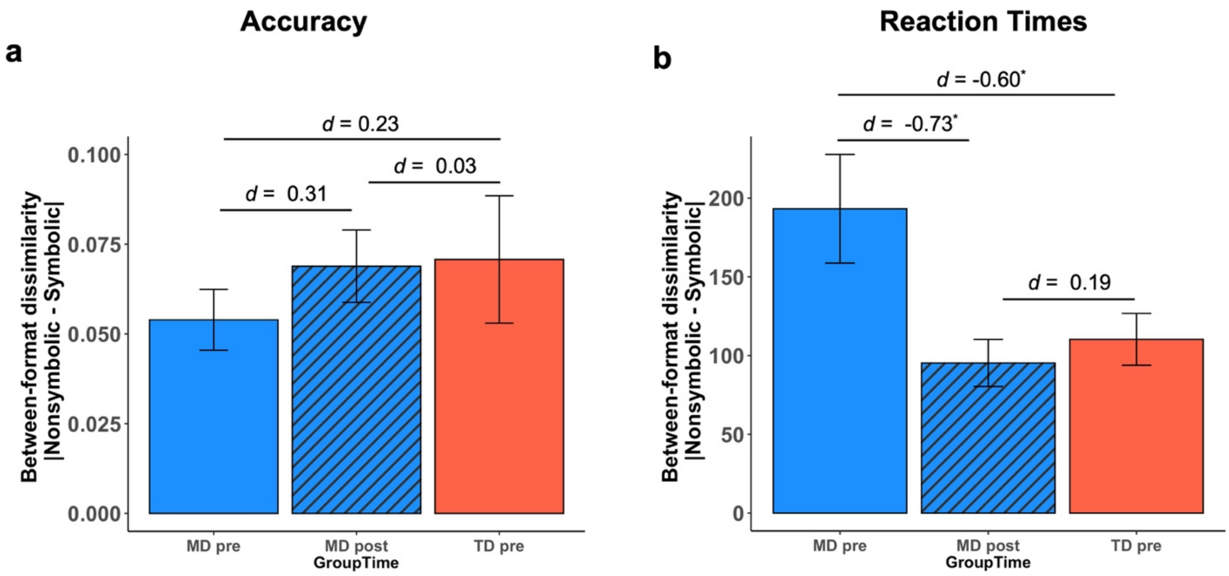

Supplementary Figure 1. CFN tutoring normalized weak cross-format similarity in behavioral performance between nonsymbolic and symbolic number formats.

Between-format dissimilarity was measured by the absolute difference in behavioral performance between nonsymbolic and symbolic number comparison tasks (|Nonsymbolic – Symbolic|). Higher scores represented higher between-format dissimilarity (or lower cross-format similarity) in behavioral performance. **(a)** Between-format dissimilarity measured by accuracy. Effect sizes indicate that group difference in between-format similarity at pre-tutoring ($d = 0.23$) was greater than difference in between-format similarity between the MD group at post-tutoring and the TD group at pre-tutoring ($d = 0.03$). **(b)** Between-format dissimilarity measured by reaction time. Between-format dissimilarity was significantly higher in the MD, compared to the TD, group at pre-tutoring ($p = 0.016$, Cohen's $d = -0.73$). CFN tutoring reduced between-format dissimilarity in the MD group at post-tutoring to the level of the TD group at pre-tutoring ($p = 0.503$, Cohen's $d = 0.19$). $^{*}p < 0.05$, d = Cohen's d.

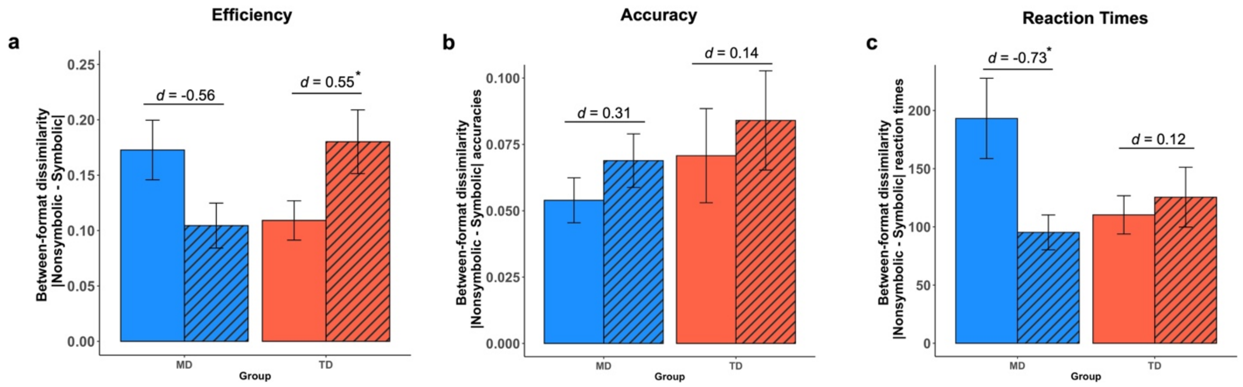

Supplementary Figure 2. CFN tutoring induced distinct patterns of changes between the MD and TD groups in cross-format similarity between nonsymbolic and symbolic number formats in behavioral performance.

Between-format dissimilarity was measured by the absolute difference in behavioral performance between nonsymbolic and symbolic number comparison tasks (|Nonsymbolic – Symbolic|). Higher scores represented higher between-format dissimilarity (or lower cross-format similarity) in behavioral performance. **(a)** Between-format dissimilarity measured by efficiency. CFN tutoring reduced between-format dissimilarity in the MD group (p = 0.064, d = -0.56) and increased between-format dissimilarity in the TD group after tutoring (p = 0.012, d = 0.55). **(b)** Between-format dissimilarity measured by accuracy. Effect sizes indicate no significant changes in between-format similarity between pre- and post-tutoring in both MD (d = 0.31) and TD group (d = 0.14). **(c)** Between-format dissimilarity measured by reaction time. CFN tutoring reduced between-format dissimilarity in the MD group (p = 0.016, d = -0.73) and no significant changes were observed in the TD group after tutoring (d = 0.12). $^*p < 0.05$, d = Cohen's d.

III. Supplementary Tables

Supplementary Table 1. Summary of previous studies of number intervention in children with or without mathematical difficulties

Study	Age		Sample Size			Training Type			Intervention Duration	Imaging
	M (yr.)	SD (mo.)	TD	TD Cntrl	MD	NonSym	Nonsym + Sym	Sym		
Wilson et al (2006) [1]	7-10 yrs				13	O	O		5-weeks, 4 days/week, 30 min/session	n/a
Opfer & Siegler (2007)[2]	8.20	7.20	61				O		3 trial blocks consisted of 10 items per each	n/a
Booth & Siegler (2008) [3]	7.20	4.80	78	27			O		1-week, 3 sessions, 10-15/session	n/a
Siegler & Ramani (2008) [4]	4.60	3.60	36				O		2-weeks, 4 sessions, 15min/session	n/a
	4.70	5.04		18						
Ramani & Siegler (2008) [5]	4.75	5.52	68[1]				O		2-weeks, 4 sessions, 15-20min/session	n/a
	4.75	4.92		56						
Siegler & Ramani (2009) [6]	4.66	5.40-5.52	59[1]				O		3-weeks, 5 sessions, 15-20min/session	n/a
	4.66	6.24	29[1]					O		
Wilson et al. (2009) [7]	5.60	4.80	53[1]			O	O		6 sessions, 20 min/session	n/a
Ramani & Siegler (2011) [8]	4.00	3.84-4.56	59				O		3-weeks, 5 sessions, 15-20min/session	n/a
	4.00	4.80	29					O		
**Kucian et al (2011) [9]**	**9.50**	**13.20**			**16**	O	O		**5-weeks, 5 sessions/week, 15 min**	**fMRI**
	**9.60**	**9.60**	**16**							
Ramini et al. (2012) [10]	4.58	6.12	34[1]				O		3-4 weeks, 6 sessions, 20-25 min/session	n/a
	4.17	6.84		28[1]						
Obersteiner et al. (2013) [11]	6.91	4.68	35			O	O		4-weeks, 30 min,10 sessions	n/a
			39			O	O	O		
			39				O	O		
				34						
Hyde et al. (2014) [12]	6.89	2.59	96			O			2 sets of practice (60 problems)	n/a

[1] Low SES children

Kuhn & Hollings (2014) [13]	9.00	8.40	20	20		O		O	3-weeks, 15 sessions, 20 min/session	n/a
Honore & Noel (2016) [14]	5.75	3.79	19	18		O			6-weeks, 10 sessions, 30 min/session	n/a
			19				O			
Sella et al (2016) [15]	5.17	8.00	23			O	O		10-weeks, 2 session/week, 20min/session	n/a
	5.00	7.00		22						
Elofsson et al. (2016) [16]	5.37	3.72	54	60			O		3 weeks, 6 sessions, 10 min/session	n/a
Maertens et al. (2016) [17]	5.44	3.48	47	63		O		O	3 weeks, 6 sessions, 10 min/session	n/a
	5.32	3.72	41				O			
Park et al.(2016) [18]	4.87	4.8	51	52		O			2-3 weeks, 10 sessions, 12min/session	n/a
Van Herwegen et al. (2017) [19]	3.77	7.20	20			O	O		5-weeks, 10min/day	n/a
	3.82	6.06		18						
**Looi et al. (2017) [20]**	**9.48**	**7.3**			**6**		tRNS		**5 weeks, 2 sessions/week, 20 min/session**	**tRNS**
					**6**		Sham			
Ramani et al. (2017) [21]	6.01	4.31	27	27			O		10 sessions, 10-15min/session	n/a
**Michels et al. (2018) [22]**	**9.50**	**8.40**			**15**	O	O		**5-weeks, 5 sessions/week, 15 min**	**fMRI**
	**9.50**	**9.60**	**16**							
Kim et al. (2018) [23]	7.70	3.60	22		24	O	O		6-weeks, 30 sessions, 24 min/session	n/a
Szkudlarek & Brannon (2018) [24]	4.57	7.32	53			O			10 sessions, 12 min/session	n/a
	4.37	7.44			[2]27					
	4.61	6.24	52					O		
	4.48	6.12			29					
	4.58	6.72		52						
	4.41	7.08			31					
Van Herwegen et al. (2018) [25]	3.62	3.99			19	O			5-weeks, 10min/day	
	3.68	4.52			19			O		
	3.77	3.91	20							
Whyte & Bull (2018) [26]	3.80	4.00	32	13			O		4 sessions, 25 min/session	n/a

[2] Among TD children, post-tested a part of children with relatively low math

Ramani et al. (2020) [27]	6.02	3.94	47				O		10 sessions, 10-15 min/session	n/a
	5.94	3.39		53						
Libertus et al. (2020) [28]	6.17	8.1	33	35		O			5 weeks, 16 sessions, 15min/ session	n/a
Vanbecelaere et al. (2020) [29]	6.37	5.04	109	223		O	O		6-weeks, 6 sessions, 50min/session	n/a
Bugden et al. (2021) [30]	9.75	7.44	53			O			6 days, 20-30min/session	n/a
	9.73	8.64		58						
Tobia et al. (2021) [31]	4.76	3.24			36	O			7-weeks, 3 sessions/week, 45 min/session	n/a
					29		O	O		
					24					
	4.77	3.6	27			O				
			23				O	O		

Abbreviations: Cntl, controls; fMRI, functional magnetic resonance imaging; M, mean; MD, mathematical difficulties or children with low math abilities; Nonsym, nonsymbolic number training; SD; standard deviation; Sym, symbolic number training; Nonsym + Sym, nonsymbolic and symbolic number training; TD, typically-developing; tRNS, transcranial random noise simulation. Bolded = studies using neuroimaging techniques.

Supplementary Table 2. Demographics and standardized assessment scores in MD and TD groups.

		MD			TD			MD vs. TD
		M	*SD*	range	*M*	*SD*	range	**p*-values
**Age**		8.36	0.64	7.4-10.0	8.06	0.47	7.3-9.1	0.123
**Gender**		8 Males, 8 Females			8 Males, 12 Females			
**WASI**	FSIQ	104.69	11.07	90-124	107.05	12.83	86-133	0.557
	VIQ	107	10.91	83-126	108.1	12.85	89-133	0.783
	PIQ	102.19	16.31	84-140	105	15.14	83-131	0.599
**WJ-III (math)**	Math fluency	85.81	3.33	80-90	102.2	8.92	92-121	<0.001
**WJ-III (reading)**	Letter word Identification	109.73	11.22	95-130	109.37	7.41	93-125	0.914
	Word attack	105.4	8.55	94-121	107.8	4.03	101-115	0.327
**AWMA (working memory)**	Digit recall	98.38	15.09	68-121	98.85	12.08	76-125	0.919
	Backward digit recall	99.38	15.34	82-143	102.8	11.98	81-122	0.470
	Word recall	87.88	17.24	64-115	89.4	16.37	64-115	0.789
	Block recall	83.62	13.87	67-109	91.15	12.65	74-111	0.103
	Spatial recall	99.94	21.2	64-128	106	14.95	83-125	0.342

Note, **p* values were obtained from two sample *t*-tests.

Supplementary Table 3. Group differences between MD and TD groups in cross-format NRS at pre-tutoring.

Region	Cluster Size	T-value	MNI coordinates in pediatric template		
			x	y	z
			**Pre-tutoring**		
TD pre > MD pre					
L LOC/IPS	607	3.87	-24	-63	47
L SPL/IPS		3.46	-30	-60	62
L SPL/IPS		3.1	-24	-54	47
L PHG	610	4.57	-15	-42	-17
L PHG		4.04	-22	-36	-15
L PHG		2.77	-17	-39	-7
L Premotor	842	3.77	-23	-16	65
L PreCG		3.72	-24	-6	64
L PreCG		3.56	-31	-9	60
R Cerebellum	672	5.82	22	-73	-23
MD pre > TD pre					
No significant clusters					

Abbreviations: IPS, Intraparietal Sulcus; SPL, Superior Parietal Lobule; L, Left; R, Right.

Supplementary Table 4. Regional-level two-sample t-test between cross-format NRS in the MD group (n = 16) at pre- or post-tutoring and cross-format NRS in the TD group (n = 20) at pre-tutoring.

ROI [MNI coordinates]	MD pre vs. TD pre			MD post vs. TD pre		
	t	***p*⁺**	**Cohen's *d***	***t***	***p*⁺**	**Cohen's *d***
L LOC/IPS [-24 -63 47]	3.51^{**}	0.003	1.16	1.52	0.432	0.51
L SPL/IPS [-30 -60 62]	2.87^{**}	0.008	0.94	0.98	0.563	0.33
L SPL/IPS [-24 -54 47]	2.97^{**}	0.007	1.00	1.97	0.290	0.63
L PHG [-15 -42 -17]	3.50^{**}	0.004	1.21	0.76	0.569	0.26
L PHG [-22 -36 -15]	3.27^{**}	0.004	1.10	0.85	0.569	0.28
L PHG [-17 -39 -7]	2.38^{*}	0.025	0.83	-0.42	0.680	-0.14
L Premotor [-23 -16 65]	3.48^{**}	0.004	1.20	0.97	0.563	0.33
L PreCG [-24 -6 64]	3.26^{**}	0.004	1.11	0.60	0.616	0.21
L PreCG [-31 -9 60]	3.57^{**}	0.003	1.21	1.41	0.432	0.50
R Cerebellum [22 -73 -23]	4.28^{***}	<0.001	1.50	2.01	0.290	0.72

Notes: ROIs were defined from a whole-brain analysis comparing cross-format NRS between the MD and TD groups at pre-tutoring (**Supplementary Table 3**). All reported coordinates are based on the pediatric template space. $^{*}p < .05$, $^{**}p < .01$, $^{***}p < .001$. $^{+}$All *p*-values are corrected for multiple comparison using FDR correction. Cohen's *d* presents absolute value. *Abbreviations*: IPS, Intraparietal Sulcus; L, Left; LOC, Lateral Occipital Cortex; PHG, Parahippocampal Gyrus; PreCG, Precentral Gyrus; Premotor, Premotor Cortex; R, Right; SPL, Superior Parietal Lobule.

Supplementary Table 5. Regional-level two-sample *t*-test between cross-format NRS in the MD group (n = 19) at pre- or post-tutoring and cross-format NRS in the TD group (n = 21) at pre-tutoring.

ROI [MNI coordinates]	MD pre vs. TD pre			MD post vs. TD pre		
	t	p^+	Cohen's d	t	p^+	Cohen's d
L LOC/IPS [-24 -63 47]	4.07^{***}	<0.001	1.28	1.33	0.332	0.42
L SPL/IPS [-30 -60 62]	3.34^{**}	0.003	1.05	1.31	0.332	0.41
L SPL/IPS [-24 -54 47]	3.78^{**}	0.002	1.20	2.24	0.155	0.70
L PHG [-15 -42 -17]	2.93^{**}	0.007	0.95	0.84	0.452	0.27
L PHG [-22 -36 -15]	2.50^{*}	0.020	0.80	0.84	0.452	0.27
L PHG [-17 -39 -7]	2.33^{*}	0.026	0.75	-0.40	0.692	-0.13
L Premotor [-23 -16 65]	4.09^{***}	<0.001	1.31	1.56	0.318	0.50
L PreCG/Premotor [-24 -6 64]	3.80^{**}	0.002	1.20	0.88	0.452	0.28
L PreCG/Premotor [-31 -9 60]	4.16^{***}	<0.001	1.32	2.01	0.180	0.65
R Cerebellum [22 -73 -23]	3.65^{**}	0.002	1.19	2.39	0.155	0.78

Notes: ROIs were defined from a whole-brain analysis comparing cross-format NRS between the MD and TD groups at pre-tutoring (**Supplementary Table 3**). All reported coordinates are based on the pediatric template space. $^{*}p < .05$, $^{**}p < .01$, $^{***}p < .001$. $^{+}$All p-values are corrected for multiple comparison using FDR correction. Cohen's d presents absolute value. Abbreviations: IPS, Intraparietal Sulcus; L, Left; LOC, Lateral Occipital Cortex; PHG, Parahippocampal Gyrus; PreCG, Precentral Gyrus; Premotor, Premotor Cortex; R, Right; SPL, Superior Parietal Lobule.

Supplementary Table 6. Brain regions showing significant interaction between group (MD vs. TD) and time (pre- vs. post- tutoring) on cross-format NRS in Group X Time ANCOVA.

Region	Cluster Size	T-value	MNI coordinates		
			x	y	z
MD (post>pre) > TD (post>pre)					
L Cerebellum	882	4.61	-26	-63	-29
L Occipital Fusiform gyrus		3.35	-36	-64	-22
L Cerebellum		3.34	-20	-70	-28
L PHG	571	4.38	-20	-38	-16
L Cerebellum		4.1	-16	-46	-19
TD (post>pre) > MD (post>pre)					
No significant clusters					

Abbreviations: L, Left; PHG, Parahippocampal Gyrus; R, Right.

Supplementary Table 7. Brain regions showing significant main effect of group (MD vs. TD) on cross-format NRS in Group X Time ANCOVA.

Region	Cluster Size	T-value	MNI coordinates		
			x	y	z
TD > MD					
R Fusiform gyrus	550	4.71	22	-80	-23
R Cerebellum		4.14	21	-72	-22
R Cerebellum		4.08	30	-77	-25
R SPL/IPS	766	5.24	27	-62	63
R SPL/IPS		3.89	21	-66	58
R SPL/IPS		3.32	39	-51	59
L SPL/IPS	801	4.37	-37	-51	51
L SPL/IPS		4.31	-38	-43	55
MD > TD					
No significant clusters					

Abbreviations: IPS, Intraparietal Sulcus; L, Left; R, Right; SPL, Superior Parietal Lobule.

Supplementary Table 8. Regional-level paired t-test between cross-format NRS at pre- and post-tutoring in each MD (n = 16) and TD group (n = 20).

ROI [MNI coordinates]	MD pre vs. post			TD pre vs. post		
	t	***p*⁺**	***d***	***t***	***p*⁺**	***d***
L Cerebellum [-26 -63 -29]	3.49*	0.015	0.93	-2.36*	0.036	-0.63
L Fusiform gyrus [-36 -64 -22]	1.81	0.091	0.57	-2.41*	0.036	-0.67
L Cerebellum [-20 -70 -28]	1.95	0.086	0.68	-2.39*	0.036	-0.54
L PHG [-20 -38 -16]	2.26	0.065	0.78	-2.81*	0.036	-0.82
L Cerebellum [-16 -46 -19]	2.42	0.065	0.84	-1.43	0.168	-0.31

Notes: ROIs were defined from a whole-brain Group X Time ANCOVA analysis showing significant interaction effect (**Supplementary Table 6**). All reported coordinates are based on the pediatric template space. $^{*}p < .05$. $^{+}$All *p*-values are corrected for multiple comparison using FDR correction. Cohen's *d* presents absolute value. Abbreviations: L, Left; PHG, Parahippocampal Gyrus; R, Right.

Supplementary Table 9. Regional-level paired t-test between cross-format NRS at pre- and post-tutoring in each MD (n = 19) and TD group (n = 21).

ROI [MNI coordinates]	MD pre vs. post			TD pre vs. post		
	t	p^+	*d*	*t*	p^+	*d*
L Cerebellum [-26 -63 -29]	3.49*	0.015	0.85	-2.46*	0.034	-0.63
L Fusiform gyrus [-36 -64 -22]	0.67	0.510	0.19	-2.61*	0.034	-0.7
L Cerebellum [-20 -70 -28]	1.38	0.233	0.46	-2.39*	0.034	-0.53
L PHG [-20 -38 -16]	1.69	0.178	0.54	-2.90*	0.034	-0.82
L Cerebellum [-16 -46 -19]	1.82	0.178	0.66	-1.63	0.119	-0.35

Notes: ROIs were defined from a whole-brain Group X Time ANCOVA analysis showing significant interaction effect (**Supplementary Table 6**). All reported coordinates are based on the pediatric template space. $^*p < .05$. $^+$All *p*-values are corrected for multiple comparison using FDR correction. Cohen's *d* presents absolute value. Abbreviations: L, Left; PHG, Parahippocampal Gyrus; R, Right.

VI. Supplementary References

1 Wilson, A. J., Revkin, S. K., Cohen, D., Cohen, L. & Dehaene, S. An open trial assessment of "The Number Race", an adaptive computer game for remediation of dyscalculia. *Behavioral and Brain Functions*, 16 (2006).

2 Opfer, J. E. & Siegler, R. S. Representational change and children's numerical estimation. *Cognitive psychology* **55**, 169-195 (2007).

3 Booth, J. L. & Siegler, R. S. Numerical magnitude representations influence arithmetic learning. *Child development* **79**, 1016-1031 (2008).

4 Siegler, R. S. & Ramani, G. B. Playing linear numerical board games promotes low-income children's numerical development. *Developmental science* **11**, 655-661 (2008).

5 Ramani, G. B. & Siegler, R. S. Promoting broad and stable improvements in low-income children's numerical knowledge through playing number board games. *Child development* **79**, 375-394 (2008).

6 Siegler, R. S. & Ramani, G. B. Playing linear number board games—but not circular ones—improves low-income preschoolers' numerical understanding. *Journal of educational psychology* **101**, 545 (2009).

7 Wilson, A. J., Dehaene, S., Dubois, O. & Fayol, M. Effects of an adaptive game intervention on accessing number sense in low-socioeconomic-status kindergarten children. *Mind, Brain, and Education* **3**, 224-234 (2009).

8 Ramani, G. B. & Siegler, R. S. Reducing the gap in numerical knowledge between low- and middle-income preschoolers. *Journal of Applied Developmental Psychology* **32**, 146-159 (2011). https://doi.org/https://doi.org/10.1016/j.appdev.2011.02.005

9 Kucian, K. *et al.* Mental number line training in children with developmental dyscalculia. *NeuroImage* **57**, 782-795 (2011). https://doi.org/10.1016/j.neuroimage.2011.01.070

10 Ramani, G. B., Siegler, R. S. & Hitti, A. Taking it to the classroom: Number board games as a small group learning activity. *Journal of educational psychology* **104**, 661 (2012).

11 Obersteiner, A., Reiss, K. & Ufer, S. How training on exact or approximate mental representations of number can enhance first-grade students' basic number processing and arithmetic skills. *Learning and Instruction* **23**, 125-135 (2013).

12 Hyde, D. C., Khanum, S. & Spelke, E. S. Brief non-symbolic, approximate number practice enhances subsequent exact symbolic arithmetic in children. *Cognition* **131**, 92-107 (2014).

13 Kuhn, J.-T. & Holling, H. Number sense or working memory? The effect of two computer-based trainings on mathematical skills in elementary school. *Advances in cognitive psychology* **10**, 59 (2014).

14 Honore, N. & Noel, M. P. Improving Preschoolers' Arithmetic through Number Magnitude Training: The Impact of Non-Symbolic and Symbolic Training. *PLoS One* **11**, e0166685 (2016). https://doi.org/10.1371/journal.pone.0166685

15 Sella, F., Tressoldi, P., Lucangeli, D. & Zorzi, M. Training numerical skills with the adaptive videogame "The Number Race": A randomized controlled trial on preschoolers. *Trends in Neuroscience and Education* **5**, 20-29 (2016).

16 Elofsson, J., Gustafson, S., Samuelsson, J. & Träff, U. Playing number board games supports 5-year-old children's early mathematical development. *The Journal of Mathematical Behavior* **43**, 134-147 (2016). https://doi.org/https://doi.org/10.1016/j.jmathb.2016.07.003
17 Maertens, B., De Smedt, B., Sasanguie, D., Elen, J. & Reynvoet, B. Enhancing arithmetic in pre-schoolers with comparison or number line estimation training: Does it matter? *Learning and Instruction* **46**, 1-11 (2016). https://doi.org/https://doi.org/10.1016/j.learninstruc.2016.08.004
18 Park, J., Bermudez, V., Roberts, R. C. & Brannon, E. M. Non-symbolic approximate arithmetic training improves math performance in preschoolers. *Journal of experimental child psychology* **152**, 278-293 (2016).
19 Van Herwegen, J., Costa, H. M. & Passolunghi, M. C. Improving approximate number sense abilities in preschoolers: PLUS games. *School Psychology Quarterly* **32**, 497 (2017).
20 Looi, C. Y. *et al.* Transcranial random noise stimulation and cognitive training to improve learning and cognition of the atypically developing brain: A pilot study. *Scientific Reports* **7**, 4633 (2017).
21 Ramani, G. B., Jaeggi, S. M., Daubert, E. N. & Buschkuehl, M. Domain-specific and domain-general training to improve kindergarten children's mathematics. *Journal of Numerical Cognition* **3**, 468-495 (2017).
22 Michels, L., O'Gorman, R. & Kucian, K. Functional hyperconnectivity vanishes in children with developmental dyscalculia after numerical intervention. *Developmental Cognitive Neuroscience* **30**, 291-303 (2018). https://doi.org/10.1016/j.dcn.2017.03.005
23 Kim, N., Jang, S. & Cho, S. Testing the efficacy of training basic numerical cognition and transfer effects to improvement in children's math ability. *Frontiers in Psychology* **9**, 1775 (2018).
24 Szkudlarek, E., Park, J. & Brannon, E. M. Failure to replicate the benefit of approximate arithmetic training for symbolic arithmetic fluency in adults. *Cognition* **207**, 104521 (2021).
25 Van Herwegen, J., Costa, H. M., Nicholson, B. & Donlan, C. Improving number abilities in low achieving preschoolers: Symbolic versus non-symbolic training programs. *Research in developmental disabilities* **77**, 1-11 (2018).
26 Whyte, J. C. & Bull, R. Number games, magnitude representation, and basic number skills in preschoolers. *Developmental psychology* **44**, 588 (2008).
27 Ramani, G. B. *et al.* Racing dragons and remembering aliens: Benefits of playing number and working memory games on kindergartners' numerical knowledge. *Developmental science* **23**, e12908 (2020).
28 Libertus, M. E., Odic, D., Feigenson, L. & Halberda, J. Effects of visual training of approximate number sense on auditory number sense and school math ability. *Frontiers in psychology*, 2085 (2020).
29 Vanbecelaere, S. *et al.* The effects of two digital educational games on cognitive and non-cognitive math and reading outcomes. *Computers & Education* **143**, 103680 (2020).

30 Bugden, S., Szkudlarek, E. & Brannon, E. Approximate arithmetic training does not improve symbolic math in third and fourth grade children. *Trends in Neuroscience and Education* **22**, 100149 (2021).

31 Tobia, V., Bonifacci, P. & Marzocchi, G. M. Symbolic versus non-symbolic training for improving early numeracy in preschoolers at risk of developing difficulties in mathematics. *Research in Developmental Disabilities* **111**, 103893 (2021).
